# Possible Associations of *NTRK2* Polymorphisms with Antidepressant Treatment Outcome: Findings from an Extended Tag SNP Approach

**DOI:** 10.1371/journal.pone.0064947

**Published:** 2013-06-04

**Authors:** Johannes M. Hennings, Martin A. Kohli, Darina Czamara, Maria Giese, Anne Eckert, Christiane Wolf, Angela Heck, Katharina Domschke, Volker Arolt, Bernhard T. Baune, Sonja Horstmann, Tanja Brückl, Torsten Klengel, Andreas Menke, Bertram Müller-Myhsok, Marcus Ising, Manfred Uhr, Susanne Lucae

**Affiliations:** 1 Max-Planck-Institute of Psychiatry, Munich, Germany; 2 Munich Cluster for Systems Neurology (SyNergy), Munich, Germany; 3 Neurobiology Laboratory for Brain Aging and Mental Health, Psychiatric University Clinics Basel, Basel, Switzerland; 4 Department of Psychiatry, University of Wuerzburg, Wuerzburg, Germany; 5 Department of Psychiatry, University of Muenster, Muenster, Germany; 6 Department of Psychiatry, The University of Adelaide, Eleanor Harrald Building, Royal Adelaide Hospital, Adelaide, Australia; University of Chicago, United States of America

## Abstract

**Background:**

Data from clinical studies and results from animal models suggest an involvement of the neurotrophin system in the pathology of depression and antidepressant treatment response. Genetic variations within the genes coding for the brain-derived neurotrophic factor (*BDNF*) and its key receptor Trkb (*NTRK2*) may therefore influence the response to antidepressant treatment.

**Methods:**

We performed a single and multi-marker association study with antidepressant treatment outcome in 398 depressed Caucasian inpatients participating in the Munich Antidepressant Response Signature (MARS) project. Two Caucasian replication samples (*N* = 249 and *N* = 247) were investigated, resulting in a total number of 894 patients. 18 tagging SNPs in the *BDNF* gene region and 64 tagging SNPs in the *NTRK2* gene region were genotyped in the discovery sample; 16 nominally associated SNPs were tested in two replication samples.

**Results:**

In the discovery analysis, 7 *BDNF* SNPs and 9 *NTRK2* SNPs were nominally associated with treatment response. Three *NTRK2* SNPs (rs10868223, rs1659412 and rs11140778) also showed associations in at least one replication sample and in the combined sample with the same direction of effects (*P_corr_* = .018, *P_corr_* = .015 and *P_corr_* = .004, respectively). We observed an across-gene *BDNF*-*NTRK2* SNP interaction for rs4923468 and rs1387926. No robust interaction of associated SNPs was found in an analysis of BDNF serum protein levels as a predictor for treatment outcome in a subset of 93 patients.

**Conclusions/Limitations:**

Although not all associations in the discovery analysis could be unambiguously replicated, the findings of the present study identified single nucleotide variations in the *BDNF* and *NTRK2* genes that might be involved in antidepressant treatment outcome and that have not been previously reported in this context. These new variants need further validation in future association studies.

## Introduction

Despite large efforts during the last decades, antidepressant treatment efficacy in depression is still unsatisfactory [1], [2]. While antidepressants are generally effective and indispensable for the treatment of moderate and severe depression, up to two of three patients do not sufficiently respond to a first antidepressant treatment attempt [2]. Increased plasma corticosteroid levels, typically found in many patients with depression [3], may lead to a reduced trophic support of neurons and an impaired plasticity of critical brain structures involved in affective disorders [4], [5]. Stress-induced perturbation of hormonal homeostasis, and specifically elevated corticosteroid concentrations decrease the expression of hippocampal brain-derived neurotrophic factor (BDNF) [6], [7], a major mediator of neurogenesis and synaptic plasticity involved in learning and adaptive processes in the adult brain [8]. As antidepressants have been shown to reverse stress and depression induced BDNF downregulation and impaired neurogenesis [9], [10], BDNF has been implicated in recovery mechanisms from depression, which resulted in the *neurotrophin hypothesis* of depression [4], [5]. In several studies, reduced serum BDNF levels in depression and reduced hippocampal BDNF expression in mouse models of affective disorders could be reversed by various antidepressant interventions [4], [5], [11]–[13].

Apart from BDNF, the neurotrophin system comprises the nerve growth factor (NGF), neurotrophin 3 and 4/5, as well as their key receptors, the tropomycin receptor kinase (Trk) family, including the major receptor for BDNF, TrkB, encoded by the *NTRK2* gene [11]. Indeed, functional BDNF-TrkB signaling is required for behavioral effects induced by antidepressants [14] and increased *NTRK2* mRNA expression [15] and signaling [14], [16], [17] upon antidepressive interventions have been recently reported. Thus, these two genes are in the focus of this study.

Up to now, pharmacogenetic studies on polymorphisms in the neurotrophin system nearly exclusively focused on the *BDNF* gene, especially the non-synonymous Val66Met (rs6265) polymorphism. Despite several attempts to replicate initial associations, many conflicting results have been reported on this polymorphism [18], [19], which could at least in part be related to population effects [20]. Despite some positive associations reported for other *BDNF* and *NTRK2* SNPs [21], [22], previous pharmacogenetic studies did not find a major impact of *BDNF* and *NTRK2* polymorphisms on antidepressant treatment including a German [23] and the STAR*D sample [24]. Presently there are no SNPs of the neurotrophin system that have been unambiguously shown to be associated with treatment response in depression.

We extended the previous approaches by testing pharmacogenetic associations not only for variants in the *BDNF* gene but also for variations in its main receptor gene, *NTRK2,* in depressed German inpatients participating in the Munich Antidepressant Response Signature (MARS) project, which is a naturalistic study dedicated to identify predictors of antidepressant treatment outcome. We selected 82 tagging SNPs covering 100% of *BDNF* variants with a minor allele frequency (MAF) ≥0.01 and 92.8% of *NTRK2* variants with a MAF ≥0.1 based on HapMap project data. We tested all nominally significant SNPs out of the discovery analysis in 2 replication samples, resulting in a total sample size of 894 patients. In addition to our single-marker analysis we performed multi-marker approaches using a haplotype as well as a two-way interaction analysis.

## Methods

### Ethics Statement

The study was approved by the Ethics Committees of the Medical Faculties at the Ludwig Maximilians University, Munich, Germany, and at the University of Muenster, Muenster, Germany, respectively. Written informed consent was obtained from all subjects, and the study was carried out in accordance with the latest revision of the Declaration of Helsinki.

### Sample Description

398 Caucasian inpatients (56.0% females, mean age 49.1±14.4 (SD) years) that participated in the MARS project (http://www.mars-depression.de) were included within 5 days after admission to the clinic for the treatment of an acute depressive episode, as described in detail previously [1]. Depressive symptoms were rated weekly with the 21-items version of the Hamilton Rating Scale for Depression (HAM-D) and all patients were at least moderately severe depressed at inclusion (HAM-D ≥14). Two replication samples were defined: (1.) 249 newly recruited participants of the MARS project (MARS replication; 51.4% females, mean age 48.0±13.6 (SD) years), and (2.) 247 of 340 Caucasian inpatients with an HAM-D ≥14 at inclusion recruited in a pharmacogenetic study at the University of Muenster, Westphalia, Germany [23], [25] (Muenster replication; 59.1% females, mean age 49.2±14.9 (SD) years; see Table 1). MARS patients (*N* = 647) suffered from a single major depressive episode (single MDE; 32.9%), recurrent depression (RD; 56.1%) or bipolar depression (BP; 11.0%). Among MARS bipolar patients, 42.6% (29 of 68) were subclassified as bipolar type 1 and 57.4% (39 of 68) as bipolar type 2. All patients received antidepressant treatment according to the choice of the attending doctor with antidepressant dosages adjusted according to therapeutic plasma level ranges. In accordance with previous studies from the MARS project [1], [26]–[30], response was defined as an at least 50% reduction after five weeks of the HAM-D score at admission. Remission at discharge was defined as a HAM-D <10 (mean duration of hospital stay: 11.5±7.8 (SD) weeks).

**Table 1:** Sample characteristics.

	All patients	MARS Discovery	MARS Replication	Muenster Replication	
	*N* = 894	*N* = 398	*N* = 249	*N* = 247	*P* a
Age, mean (SD), y	48.9 (14.3)	49.1 (14.4)	48.0 (13.6)	49.2 (14.9)	.58
Female gender, No. (%)	497 (55.6)	223 (56.0)	128 (51.4)	146 (59.1)	.22
Unipolar depression, No. (%)	780 (87.2)	345 (86.7)	231 (92.8)	204 (82.6)	**<.01**
HAM-D at admission, mean (SD)	25.9 (6.6)	24.4 (6.4)	27.1 (5.8)	24.0 (7.2)	**<.001**
HAM-D at discharge, mean (SD)	7.6 (6.0)	9.1 (5.9)	7.1 (6.2)	5.8 (5.2)	**<.001**
Duration of hospital stay (weeks), mean (SD)	11.5 (9.2)	11.5 (7.8)	10.9 (9.0)	12.0 (11.5)	.45
Response at week 5, No. (%)	468 (52.3)	192 (50.1)	147 (63.4)	129 (53.1)	**.01**
Remission at discharge, No. (%)	581 (65.0)	204 (60.7)	172 (72.9)	202 (81.8)	**<.001**
Number of previous episodes, mean (SD)	3.01 (5.4)	2.84 (5.1)	3.51 (7.3)	2.76 (2.8)	.27
Duration of current episode (weeks), mean (SD)	38.12 (66.0)	41.37 (71.4)	32.63 (55.2)	N/A	.11
Psychotic symptoms, No. (%)	87 (13.6)	50 (12.6)	37 (15.4)	N/A	.31
Treatment resistance^b^ at admission, No. (%)	94 (16.7)	54 (15.4)	40 (18.9)	N/A	.28

a ANOVA and Chi-square, respectively, comparing the different samples. For some variables there are missing data and *N* does not equal number of total patients. Percentages are based on available data.

b Treatment resistance was defined as at least 2 antidepressant treatment attempts with an adequate duration and dosage according to Souery et al.

N/A: data not available.

### DNA Sampling and SNP Genotyping

DNA was extracted from 30 ml of EDTA blood using Puregene whole-blood DNA extraction kits (Gentra Systems, Minneapolis, USA). Using the Tagger software implemented in the HapMap project browser (de Bakker, *et al* 2005), we retrieved tagging SNPs for the longest *BDNF* isoform (NM_170731, 66.86 kb; 18 SNPs) and the full-length *NTRK2* gene (NM_006180, 355.04 kb; 64 SNPs), flanked by additional 20 kb of both 5′ and 3′ sequences according to Human HapMap Project Phase I and II data for the CEU population, leading to 100% and 92.8% SNP marker coverage, respectively. Pair-wise r^2^ for a bin of linked SNPs was set to ≥0.8. The minor allele frequency (MAF) was set to ≥0.01 (*BDNF*) and ≥0.1 (*NTRK2*), respectively. MARS patients were genotyped using Sentrix Human-1 100 k, HumanHap 300 k and Human610 Genotyping BeadChips (Illumina Inc., San Diego, USA) and MALDI-TOF mass-spectrometer (MassArray® system), as described previously [31]. Patients form the Muenster sample were genotyped using the MALDI-TOF mass-spectrometer. In case of insufficient genotyping quality (defined as an experiment-wise call rate <.97 or significant deviation from the Hardy-Weinberg equilibrium, HWE), melting curve analysis using real-time PCR was performed (rs11602246, rs1659412, rs2049046, rs1491850 in the Muenster replication sample; rs11140778 in the MARS replication sample). SNPs that entered analysis did not significantly deviate from HWE (Table 2, S1, S2) using a Bonferroni-corrected.05 level of significance (α = .05/82 = 6.1×10^−4^).

**Table 2:** Nominally associated *BDNF* and *NTRK2* SNPs in the MARS discovery sample.

SNP	Gene	Function^a^	Minor/major allele	Genotype			MAF	HWE *P* b	*P* c (allelic)	*P* c (genotypic)
				1/1	1/2	2/2				
rs2049048	*BDNF*	5'	T/C	277	110	11	.17	>.99	**.014**	**.008**
rs1491850	*BDNF*	5'	C/T	122	189	87	.46	.42	**.009**	**.03**
rs4923468	*BDNF*	intron	A/C	388	10	0	.01	>.99	**.01**	>.99
rs2049046	*BDNF*	intron	A/T	133	175	90	.45	.03	**4.88×10^−5^**	**4.66×10^−4^**
rs6265	*BDNF*	Val66Met	A/G	242	127	29	.23	.04	**.04**	**.07**
rs11602246	*BDNF*	3'	G/C	322	69	7	.10	.17	**.009**	**.034**
rs11030094	*BDNF*	3'	A/G	140	178	80	.42	.10	**1.52×10^−4^**	**6.05×10^−4^**
rs10868223	*NTRK2*	5'	T/C	308	82	8	.12	.35	**.03**	.11
rs1659412	*NTRK2*	5'	C/T	320	63	7	.10	.08	**.02**	.08
rs1662695	*NTRK2*	intron	C/T	301	93	4	.13	.37	**.04**	.11
rs11140778	*NTRK2*	intron	T/A	247	133	18	.21	>.99	**.004**	**.01**
rs2277193	*NTRK2*	intron	C/T	205	155	35	.28	.46	**.02**	.08
rs1948308	*NTRK2*	intron	C/T	123	202	73	.44	.61	**.008**	**.008**
rs17418241	*NTRK2*	intron	T/C	331	65	2	.09	.75	**.04**	.09
rs1387926	*NTRK2*	intron	A/G	301	93	3	.12	.17	**.047**	.08
rs1490402	*NTRK2*	3'	G/A	295	98	5	.14	.40	**.009**	**.01**

a According to dbSNP build 132.

b Uncorrected *P* values for the deviation from Hardy-Weinberg-Equilibrium; note that no *P* value exceeded the corrected (82 SNPs, Bonferroni) threshold of p<6.1×10^−4^.

c Nominal *P* values for associations with response after 5 weeks (strongest phenotype in the discovery sample).

### Protein Analysis

Morning fasting serum was available in 93 MARS patients at admission and after 6 weeks of treatment (mean 6.2+/−1.6 weeks) as well in 97 age- and gender-matched healthy controls derived from a German sample recruited at the Max Planck Institute of Psychiatry, Munich. Among patients, 47 were classified as remitters and 65 as responders according to the above mentioned criteria. Serum total BDNF levels were assessed with an enzyme-linked immunoabsorbant assay (ELISA) kit (Promega BDNF Emax®, Madison, Wis.). All measurements were performed in duplicates and mean values were taken for further analysis (mean difference between unicates was 6.5% +/−6.1 SD).

### Power Calculation

The CaTS Power Calculator for Genetic Studies [32] was used for power calculation using a two-stage design. The experiment-wise alpha error was set to 6.1×10^−4^ according to a Bonferroni-correction for 82 investigated SNPs. Given a predicted response rate to antidepressant treatment of 50% in an inpatient setting after 5 weeks [1], we calculated that a total sample size of *N* = 880 is sufficient to achieve a power of at least 90% (additive model) to detect genetic effects in a joint analysis of the discovery and replication samples assuming a relative risk of 1.40 or larger for SNPs with an allele frequency of at least 10%. If a multiplicative model is assumed, as it is proposed for complex diseases [33], a power of 93% can be expected. We can conclude from this power analysis that the combined sample size of the present study (N = 894) should be sufficient for detecting small to moderate effects of the investigated polymorphisms.

### Statistical Analysis

Genetic association analysis with binary response variables (response at week 5 and remission at discharge) was performed by *Χ^2^* statistics using the WG-Permer software (http://www.mpipsykl.mpg.de/wg-permer) with allelic and genotypic models. For all association tests, the level of significance was set to.05, and *P* values were corrected for multiple comparisons using a resampling method as proposed by Westfall and Young [34] applying 10^5^ permutations over all performed tests (i.e., for 82 SNPs analyzed in the discovery sample and for 16 SNPs analyzed in the replication or in the combined sample). Empirical *P* values were obtained using the Monte Carlo method implemented in the WG-Permer software to approximate the exact *P* value with the given standard distribution without relying on asymptotic distributional theory [35].In case of a significant association, the Armitage’s test for trends [36] was calculated using the software provided by the Institute for Human Genetics, Munich (http://ihg2.helmholtz-muenchen.de) and confidence intervals were calculated using a log-normal distribution. Forest plots were drawn using the rmeta software package for R-2.5.0 (http://cran.r-project.org). In the replication study, we used Fisher products [37] corresponding to the geometric mean of the *P* values of the two response variables (i.e. response after 5 weeks and remission at discharge) to account for possible sample heterogeneity. We defined successful replication as a nominal association in one of the replication samples and same direction of the effect.

Group differences in demographic and baseline clinical data were compared using Pearson *χ^2^* test in case of qualitative data and with *t*-tests for independent samples in case of quantitative data. Average change during treatment outcome was evaluated with t-tests for paired samples. Additionally, an analysis of covariance was applied to evaluate the effects of treatment outcome (response, remission) on BDNF serum protein concentration with age, sex, and baseline BDNF as covariates. A logistic regression analysis was applied for evaluating SNP×protein interactions as a predictor for response or remission, respectively) using age and sex as covariates. All statistical analyses were conducted with SPSS for Windows (version 18.0, SPSS, Chicago, USA).

### Haplotype Association Testing

The phenotype showing the strongest effect in the single-marker association analysis was further investigated in a haplotype analysis using the Haploview 4.1 software [38]. In order to detect informative associations complementary to our tagging SNP approach, haplotype analysis was performed using a D’-based linkage disequilibrium (LD) map based on haplotype blocks defined according to Gabriel et al. [39] (Figure S1). Analysis was performed in the discovery sample providing sufficient marker coverage. We used 10^5^ random permutations implemented in Haploview to control for false-positive findings. Rare haplotypes (frequencies <.01) were excluded from the analysis. We report *P* values for at least nominally significant haplotypes.

### Interaction Analysis

In the combined sample, we analyzed all possible two-way interactions across both genes (7 *BDNF*×9 *NTRK2* SNPs) and between the phenotype showing the strongest effect in the single-marker association analysis using a Bonferroni-corrected.05 level of significance (α = .05/63 = 7.9×10^−3^). The interaction analysis was performed with a step-wise logistic regression using R-2.5.0 (http://cran.r-project.org). Age, sex and sample origin were included as covariates, and genotypes were coded following an allelic model to obtain maximal power.

### Linkage Disequilibrium Mapping

We used the Haploview 4.1 software to map the r^2^-based LD pattern from the CEU population (release 21). Haplotype blocks were defined according to the method of Gabriel et al. [39]. Using the SNP Annotation and Proxy Search (SNAP) program provided by the BROAD Institute (http://www.broadinstitute.org/mpg/snap), we further tested, whether the four SNPs that withstood correction for multiple testing in our combined analysis (rs2049046, rs10868223, rs1659412, rs11140778) were in gene-wise LD (within the *BDNF* and *NTRK2* gene, respectively) with at least nominally associated SNPs reported in previous studies [21]–[23], [40]–[42]. We used the 1000 Genomes Project Data set implemented in SNAP to retrieve LD informations of recently identified SNPs (e.g. rs61888800). We found only the *BDNF* SNP rs2030324, previously reported by Licinio [22], to be in high LD (r^2^ = .90) with rs2049046 of the present study. Neither for any other *BDNF* (rs7124442, rs61888800, rs908867) nor *NTRK2* SNP (rs1187362, rs1187327, rs2289656, rs2378672, rs7020204, rs2013566, rs11140793) annotated in the data set, we could identify proxies with an r^2^≥.80.

## Results

### Association Analysis with Antidepressant Treatment

We tested the association between 18 *BDNF* and 64 *NTRK2* tagging SNPs with response after week 5 and remission at discharge in the MARS study sample (*N* = 398). The strongest associations were found for response after 5 weeks and under an allelic model (Tables S1 and S2). Seven SNPs in the *BDNF* gene and nine SNPs in the *NTRK2* gene were nominally associated with the strongest associated phenotype (response after 5 weeks). In the *BDNF* gene region, four SNPs were located in untranslated regions (two 5′ and two 3′ UTRs), two within introns, and one SNP (rs6265) within an exon resulting in a valine-methionine amino acid exchange (Val66Met, Table 2 and Figure S2a,b). The strongest associations were found for rs2049046 (intronic; *P* = 4.9×10^−5^) and rs11030094 (3′ UTR; *P* = 1.5×10^−4^). In the sample analyzed, both SNPs were in strong LD (r^2^ = .92). Within the *NTRK2* gene, the intronic SNP rs11140778 (*P* = 4.12×10^−3^) showed the strongest association with response after 5 weeks.

### Replication Studies

In order to replicate these findings we genotyped the 16 SNPs showing nominally significant associations for the strongest phenotype (response after 5 weeks) in the MARS discovery sample in two replication samples. Although all patients fulfilled the same inclusion criteria (at least moderate depressive episode: HAM-D≥14) and did not differ in baseline demographics, there were significant differences in baseline depression severity and response to antidepressant treatment between the three samples (Table 1). In particular, patients in the Muenster replication sample had a lower HAM-D at baseline compared to the discovery sample (*P*<.001; Bonferroni *post-hoc* test). Both replication samples had lower HAM-D scores at discharge than the discovery sample (*P*<.001, each; Bonferroni *post-hoc* test). Highest remission rates at discharge were observed in the Muenster sample, whereas highest response rates after week 5 were found in the MARS replication sample. To better account for this sample heterogeneity, we used Fisher Products [37] of these two phenotypes (i.e. response after 5 weeks and remission at discharge) in subsequent analyses of the replication samples (referred to as ‘outcome’ if not otherwise specified).

**Figure 1:**
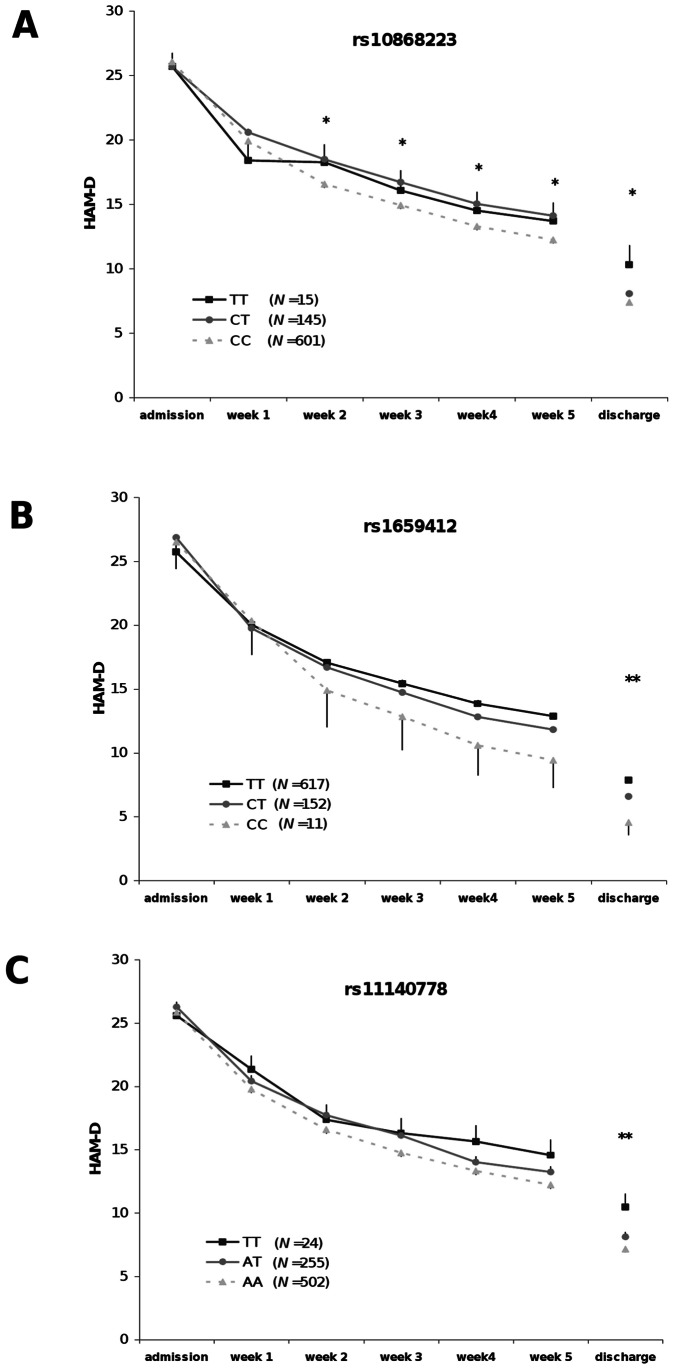
Development of HAM-D values during antidepressive treatment depending on the rs10868223 (A), rs1659412 (B) and rs11140778 (C) genotype (combined sample). Repeated measurements (Greenhouse-Geisser, age and sex as covariates) revealed significant interaction effects for rs10868223 (*P* = .007) and rs1659412 (*P* = .012), but not for rs11140778 (*P* = .645). Stars indicate significant between-subjects differences at different time points (*, p<.05; **, p<.01; GLM with age and sex as covariates). Error bars are standard errors of the means.

**Figure 2:**
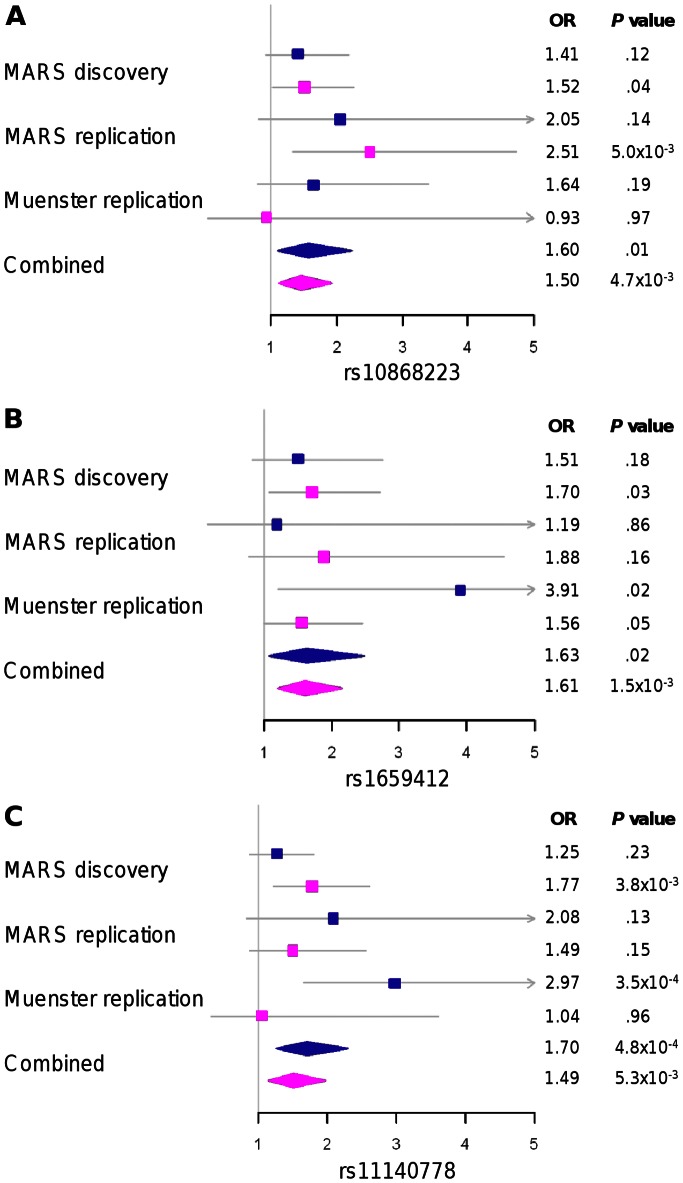
Forest plots for the three replicated *NTRK2* SNPs rs10868223 (A), rs1659412 (B) and rs11140778 (C). For each sample, odds ratios and *P* values calculated with the Armitage’s test are indicated separately for the two phenotypes remission at discharge (blue boxes) and response after week 5 (pink boxes). Diamonds were used for the combined sample (*N* = 894).

Treatment outcome was tested in replication samples under the allelic model, which showed the strongest associations in the discovery sample. Of the 16 nominally associated SNPs in the discovery sample, we found one *BDNF* SNP (rs11602246; *P* = .01) and one *NTRK2* SNP (rs10868223; *P* = .009) to be nominally associated with outcome in the MARS replication sample (*N* = 249). In the Muenster replication sample, the three *NTRK2* SNPs rs1659412, rs1662695 and rs11140778 showed nominal associations (*P* = .01; *P* = .03; *P* = .003, respectively). In the combined analysis of all patients across the three samples we found the SNPs rs2049046 (*BDNF*; *P_corr_* = .021), rs10868223 (*NTRK2*; *P_corr_* = .018), rs1659412 (*NTRK2*; *P_corr_* = .015) and rs11140778 (*NTRK2*; *P_corr_* = .004) to be significantly associated with response after correction for multiple testing (Table 3, Figure 1). According to our definition of a positive replication, we could replicate the three *NTRK2* SNPs (rs10868223, rs1659412 and rs11140778) as they showed associations in at least one replication sample and had a lower *P* value in the combined analysis compared to any single sample of the study, withstanding correction for multiple testing. For these three markers, we calculated the Armitage test of trends as a measure of the effect size separately in the three different samples and for both phenotypes (Figure 2). Under the definition for a replication, the most significantly associated *BDNF* marker in the discovery sample, rs2049046 could not be replicated, although the association in the combined analysis still withstood correction for multiple testing (*P_corr_* = .02).

**Table 3:** Association with Treatment Outcome.

		MARS Discovery	MARS Replication	Muenster Replication	Combined Sample		
		*N* = 398	*N* = 249	*N* = 247	*N* = 894		
SNP	Gene	*P* a	*P* a	*P* a	*P* a	*P* b	Beneficial allele
rs2049048	*BDNF*	**.052**	.33	.05	**.019**	.24	
rs1491850	*BDNF*	**.002**	.64	.75	.06	.54	
rs4923468	*BDNF*	**.046**	*n.p.* c	.59	.60	>.99	
rs2049046	*BDNF*	**1.0×10^−5^**	.93	.82	**.002**	**.021**	T
rs6265	*BDNF*	.10	.17	.64	.07	.60	
rs11602246	*BDNF*	**.014**	**.010**	.64	.32	.99	
rs11030094	*BDNF*	**9.0×10^−5^**	.70	.73	**.016**	.19	
rs10868223	*NTRK2*	**.039**	**.009**	.49	**.002**	**.018**	C
rs1659412	*NTRK2*	**.041**	.41	**.010**	**.001**	**.015**	C
rs1662695	*NTRK2*	**.077**	.72	**.032** d	**.038**	.42	
rs11140778	*NTRK2*	**.014**	.10	**.003**	**1.8×10^−4^**	**.004**	A
rs2277193	*NTRK2*	**.13**	.09	.60	.61	>.99	
rs1948308	*NTRK2*	**.009**	.08	1.00	**.013**	.19	
rs17418241	*NTRK2*	**.025**	.29	.72	.54	>.99	
rs1387926	*NTRK2*	**.021**	.36	.76	.30	.99	
rs1490402	*NTRK2*	**.004**	.21	.91	.08	.70	

a Empirical *P* values for the associations with treatment outcome (FPM analysis) under an allelic model.

b *P* value, permutation-based correction for multiple testing (16 SNPs).

c Not polymorphic.

d Note that this *P* value would not fulfill a more conservative threshold of alpha = .025 correcting for the fact that two replication samples have been analyzed.

As the results of single SNP associations might be confounded by combining patients with bipolar and unipolar depression, we reanalyzed the combined sample for unipolar depressed patients only (*N* = 780; 16 SNPs). Again, rs10868223, rs1659412 and rs11140778 showed the strongest association with outcome withstanding correction for multiple testing (*P_corr_* = .009, *P_corr_* = .04 and *P_corr_* = .012, respectively).

### Medication- and Gender-specific Associations

Stratifying the patients in the combined sample according to their antidepressant medication (tricyclic antidepressants, TCA; serotonin-noradrenaline reuptake inhibitors, SNRI;noradrenergic specific-serotonergic antidepressants, NASSA; noradrenaline reuptake inhibitors, NARI; monoaminoxidase inhibitors, MAOI and others) revealed SSRI-specific associations for rs2049046 (*BDNF*) and rs11140778 (*NTRK2*). Co-medication of mood stabilizers, benzodiazepines and lithium did not reveal genotype-dependent differences in prescription rates for these SNPs. Gender-specific effects were observed for rs11140778 (males) and rs1659412 (females) (Tables S3–S5; Document S1).

### Haplotype Association Testing

**Figure 3:**
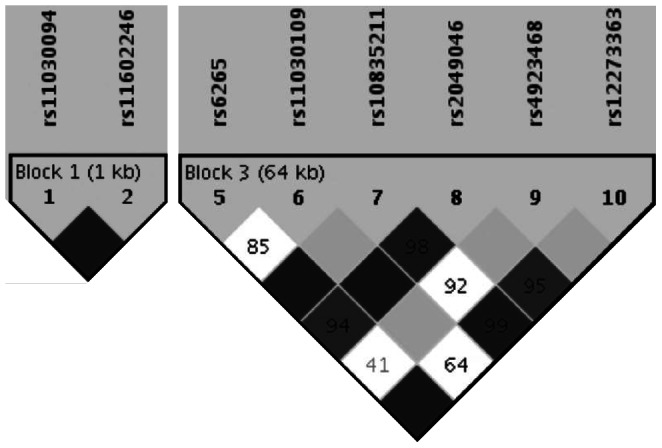
Significant haplotype blocks, both located in the *BDNF* gene, which showed association with response after 5 weeks (best phenotype of the single marker analysis). Note, that for optimal gene coverage, haplotype analysis was performed in the discovery sample only (*N* = 398, 82 SNPs).

We performed a haplotype-based association analysis to test for informative associations complementary to our single marker approach using the discovery sample providing sufficient marker coverage (82 SNPs, best phenotype of the single marker analysis in this sample (response after week 5), allelic model). Nine of twenty D’-based haplotype blocks (2 in the *BDNF* and 7 in the *NTRK2* gene, respectively; Figure S1) contained nominally significant haplotypes, most of them including SNPs that had been nominally associated in the single marker analysis of both, the discovery as well as the combined sample (Figure 3, Table S6). The GC haplotype of block 1 (rs1030094, rs11602246) and the GGGACT haplotype of block 3 (r6265, rs11030109, rs10835211, rs2049046, rs4923468 and rs12273363), both within the *BDNF* gene, showed a significant association withstanding a permutation-based correction for multiple testing (*P*_corr_ = 7.4×10^−3^ and *P*_corr_ = 7.3×10^−3^, respectively).

### Interaction Analysis

We performed gene-wise logistic regression for each pair of 7 *BDNF* and 9 *NTRK2* SNPs genotyped in the combined sample, including age, sex and sample origin as covariates. Given a Bonferroni-based threshold of significance for 7×9 = 63 statistical tests (α = 7.9×10^−3^), the interaction of the *BDNF* SNP rs4923468 with the *NTRK2* SNP rs1387926 showed a significant association with outcome (*P* = 4.6×10^−3^). No other pairs of *BDNF* and *NTRK2* SNPs showed associations.

### BDNF Protein Analysis

In order to evaluate the potential functional impact of the associated polymorphisms, we measured BDNF serum protein concentration in 93 unselected MARS patients with available blood samples at admission and after antidepressant treatment. Compared to 97 age- and sex-matched healthy controls, no difference in baseline BDNF concentration could be observed (22.13 ng/ml +/−9.98 SD (patients) *vs.* 22.49 ng/ml +/−9.74 SD (controls); *P* = .803). Also during antidepressant treatment, no change in average BDNF levels was found (22.13 ng/ml +/−9.98 SD *vs.* 23.19 ng/ml +/−10.00 SD; *P* = .258, paired t-test). However, when considering antidepressant treatment outcome in terms of remission status, we observed a significant effect on the BDNF concentration after treatment (*P* = .024) with higher BDNF concentration levels in remitters. No effects were detected for response status (*P* = .279). When testing the moderating effects of *NTRK2* SNPs showing a replicated effect on treatment outcome, we observed a genotype×protein (after treatment) interaction for rs10868223 with remission (*P* = .019) and for rs11140778 with response (*P* = .038) (see Figure S3). These effects were significant at the nominal level, not surviving correction for multiple testing.

Further, interrogating a publicly available expression quantitative trait loci (eQTL) database (GENEVAR; [43]) for the three *NTRK2* SNPs in the combined analysis with transcripts of either *BDNF* or *NTRK2*, we found nominal associations for rs11140778 and rs10868223 with two *BDNF* transcripts (GI_34106709-A, and GI_34106708-I; *P* = .03 and *P* = .03) not withstanding correction for multiple testing.

## Discussion

This pharmacogenetic study investigated the association of 18 *BDNF* and 64 *NTRK2* tagging SNPs with antidepressant treatment outcome in a representative sample of Caucasian inpatients. We tested all 16 nominally significant SNPs of the discovery analysis in 2 replication samples, resulting in a total sample size of 894 patients.

We found 3 *NTRK2* SNPs (rs10868223, rs1659412 and rs11140778) showing associations in the discovery and at least one replication sample as well as in the combined sample of 894 patients with a lower *P* value as compared to the discovery sample and withstanding correction for multiple testing. None of these *NTRK2* markers has been investigated in pharmacogenetic studies so far. Nevertheless, the functional impact of these non-coding SNPs remains unclear as robust association could neither be found in our protein analysis nor in a publicly available eQTL database.

*NTRK2* polymorphisms have so far been studied for association with childhood-onset mood disorders [40], [44], Alzheimer’s disease [45], suicidality [31], [46] and antidepressant response [21], [24], [47]. Dong and colleagues [21] found two *NTRK2* coding SNPs (rs2289657 and rs56142442) to be associated with response in a Mexican American MDD sample (*N* = 272), whereas association of *NTRK2* SNPs was neither found in the GENDEP [47] nor the STAR*D sample [24]. The significant *NTRK2* SNPs found in the present study are located 5′ of the transcriptional unit (rs10868223 and rs1659412) and in an intron (rs11140778). They are not in LD with previously reported *NTRK2* SNPs. In addition, as previous studies did not tag regions far outside the coding regions, rs10868223 and rs1659412 could not have been detected so far.

Similarly to previous studies that report interactive *BDNF*×*NTRK2* interaction on the SNP level for geriatric depression [48] and suicidality [31], [46], we found a *BDNF*×*NTRK2* SNP interaction for antidepressant treatment outcome in the present study. The findings of our interaction study have to be interpreted cautiously as only nominally associated SNPs from the initial sample entered the analysis and we corrected the p-value only for this subset of SNPs. Future studies, including SNP×SNP and SNP×protein analyses, need to substantiate these initial findings and may help elucidating the complex interplay of BDNF and its main receptor TrkB.

It has been suggested that various antidepressants may act differentially on the neurotrophin system [49] and pharmacogenetic associations may be restricted to specific drug treatments [22]. In the *BDNF* gene, Dwivedi et al. found a desipramine-specific increase in exons I and III mRNA in both the frontal cortex and hippocampus, whereas fluoxetine increased only exon II mRNA in the hippocampus [49]. Similarly, we found a differential pattern of associated SNPs dependent on the type of antidepressant in our study. In particular, the intronic *BDNF* SNP rs2049046 and the *NTRK2* SNP rs11140778, both significantly associated in the combined sample, may exert their effects on response via SSRI-mediated mechanisms. We found that the strong association of rs2049046 in the discovery sample is related to SSRI treated patients only. Interestingly, Domschke et al. [23] described a SSRI-specific association for the *BDNF* SNP rs7124442, and most previous studies showing pharmacogenetic associations with *BDNF* included SSRI treated patients only [19], [50]–[52]. However, Licinio et al. [22] described an intronic *BDNF* SNP to be nominally associated with response in a desipramine treated, but not a fluoxetine treated subgroup of Mexican Americans. Our own results do not contradict the hypothesis of medication-specific effects of *BDNF* polymorphisms upon treatment outcome. To further substantiate this assumption, larger samples with a randomized parallel group design are necessary.

Although our study was sufficiently powered to detect small to moderate genetic effects, we could not resolve the ambiguity of pharmacogenetic findings for *BDNF*. Several clinical and preclinical studies have assigned a major impact of BDNF in the pathophysiology of depression and its recovery mechanisms, while pharmacogenetic and case-control studies on *BDNF* polymorphisms have produced conflicting results [19], [22]–[24], [41], [47], [50]–[56]. In particular, a recent meta-analysis including a total of 2812 MDD patients and 10843 controls did not find an association of the functional Val66Met (rs6265) polymorphism with unipolar depression in the total sample, but in a separate analysis of male participants [57]. Similarly, no consistent case-control association could be demonstrated in bipolar depression [42], [58]–[61]. Considerable global population diversity of *BDNF* allele and haplotype frequencies have been held responsible for these inconsistencies across studies [20].

In an extensive exonic analysis including novel sequence variants in a Mexican American sample, Licinio et al. [22] detected eight nominally significant markers associated with response. Interestingly, one of them, rs2030324 is located in the same LD block as rs2049046 in the CEU population (SNP’s pair-wise r^2^ = 0.9) that showed significant association in the combined sample of the present analysis. Nevertheless, the SNP was not associated in our replication samples. Using different *BDNF* SNPs, one of our replication samples was previously analyzed by Domschke and coworkers [23] who found a negative effect of the *BDNF* SNP rs7124442 TT genotype on treatment outcome (*N* = 254), particularly in anxious depression. Nevertheless, as the analysis of 10 SNPs including rs6265 in outpatients genotyped in the STAR*D sample did not show any significant association with response, the authors concluded that the *BDNF* gene does not have major impact on antidepressant treatment response and only a potential minor role in the context of melancholic depression [23]. Concerning *BDNF*, this is in line with findings from the present study.

A gender-specific stratification of our sample indicates that the strong association of the *NTRK2* SNP rs11140778 in the combined sample mainly originates from the male subgroup of patients. On the other hand, rs1659412, also significantly associated in the combined sample, appears to moderate antidepressant response predominantly in female patients. Of note, both SNPs did not even show nominal associations in the opposing gender. Gender-specific effects have been reported for *BDNF* associations with depression and antidepressant treatment [23], [57], as well as for *NTRK2* with obsessive-compulsive disorder [44]. Nevertheless, results have to be interpreted cautiously and subsamples might still have been underpowered to rule out false-negative associations.

Several limitations of the present study should be mentioned. First, although our power analysis suggested being sufficient to detect moderate genetic effects in the combined sample, the study might still have been underpowered for statistical effects in complex phenotypes like antidepressant response possibly accounting for the lack of replication of *BDNF* associations found in the discovery sample. Another issue are the multiple statistical tests performed in this study including several *post-hoc* analyses. Thus, although having corrected for the number of SNPs genotyped, reported association may still have been found just by chance. In addition, at least part of the patients have been also analyzed on genome-wide level [29] without significant association of *BDNF* and *NTRK2* polymorphisms. Nevertheless, the current study follows a candidate gene approach with selectively genotyped SNPs. Conversely, a random selection of 82 SNPs genotyped in 325 patients in the discovery sample using Human610 Genotyping BeadChips (165 responder, 160 non-responder) resulted in only two nominal significant associations (*P* = .003 and *P* = .026, respectively), not withstanding correction for 82 SNPs investigated (*P* = .118 and *P* = .874; Westfall-Young; data not shown).

Further, although statistically significant, the clinical relevance of observed genetic effects can be questioned. According to recent approaches, combining multiple genetic markers could provide more reliable and meaningful prediction of antidepressant response [29]. Nevertheless, several clinical and preclinical studies implicated pathophysiological involvement of the neurotrophins in antidepressant response [4], [5], [11]–[13]. We therefore argue, that a thorough genetic approach with extended gene coverage like in the present study can add to the understanding and characterization of the neurotrophin system in antidepressant response mechanisms.

Heterogeneity of patients is an important issue in genetic association studies and may have limited the replication of initial findings in the present study. In particular, we observed marked differences in depression severity and response rates among samples. In addition, besides unipolar depression, patients with a bipolar disorder were included, which might have impaired the homogeneity of our sample. Nevertheless, during a depressive episode, there are no pathognomonic characteristics that can reliably differentiate the two disorders and misdiagnosis of bipolar disorder as unipolar depression occurs frequently [62], [63]. Given a relatively high annual conversion rate of recurrent unipolar to bipolar disorder [64], these entities may indeed share common disease mechanisms, too. Further, neither clinical data [1], [64] nor genetic analyses [29], [65] support the view that these clinical entities must be considered separately and a re-analysis with unipolar patients only confirmed the initial findings. All patients fulfilled the same inclusion criteria and did not differ in baseline demographics, duration of hospital stay and other characteristics of the current episode. In addition, adjusting quantitative phenotype variables for sample origin did not alter the SNP associations.

Four SNPs (rs2049046, rs6265, rs9969765 and rs11140793) showed nominal deviations from the HWE (DHW) including the most significantly associated SNP in the discovery analysis (rs2049046). Using a Bonferroni-based threshold for multiple testing of the HWE can be questioned and may not be conservative enough. However, given that 82 SNPs have been genotyped in the initial sample, false positive deviations of HWE can be expected in about 4 cases for an alpha set to.05, which may have been the case in the current analysis. DHW may also rely on the underlying disease or phenotypic model itself and may thus be regarded as an indicator of a true association (discussed in [66]). Further, there was no indication for insufficient genotyping quality as a possible explanation for DHW and deviations were only slight (minimal *P* = .02) which has been reported in previous studies with associated variants (e.g. [67]).

The current study does not have a placebo arm, making it impossible to disentangle placebo-related and real pharmacological effects, and only a part of our patients was treated with only one class of antidepressant agent, increasing the risk of false-positive and false-negative findings in the medication-specific analyses. On the other hand, due to the naturalistic design of the present study allowing medication of doctor’s choice, our results are probably more generalizable and more relevant for clinical practice. In addition, still a considerable number of patients entered the medication-specific analysis and our findings are in line with previous studies that reported *BDNF* SNP associations in SSRI treated patients [19], [23], [50]–[52].

In summary, in an extended gene coverage approach, we found variants in the *NTRK2* gene that may potentially predict antidepressant treatment outcome. Consistent with previous reports, no major effect of the extensively studied *BDNF* Val66Met polymorphism with antidepressant response could be found. The functional impact of associated variants remains unclear and replications of associations in subsequent studies including functional assessments like RNA and protein measurements are warrant to further substantiate initial findings of the present study.

## Supporting Information

Figure S1 **D’-based linkage disequilibrium (LD) of the** ***BDNF*** **(left panel) and** ***NTRK2*** **(right panel) gene regions retrieved from HapMap for the CEU population (release 21).** Haplotype blocks were defined according to Gabriel et al. [39].
(TIF)

Figure S2 **R^2^-based linkage disequilibrium (LD) of the** ***BDNF*** **(A) and** ***NTRK2*** **(B) gene regions retrieved from HapMap for the CEU population (release 21).** Diamonds indicate genotyped SNPs in the discovery sample. Red diamonds represent nominally associated SNPs in the discovery sample. The exon (black boxes)-intron structures of the longest isoforms of *BDNF* (NM_170731) and *NTRK2* (NM_006180) are depicted according to dbSNP build 132. In case of *BDNF*, black boxes indicate alternative exons (I-IX) as proposed by Pruunsild, *et al* (Pruunsild, *et al* 2007), and are approximated relative to the SNPs.
(TIFF)

Figure S3 **Genotype-dependent concentration of serum BDNF levels in non-improved (dark grey bars) and improved patients (light grey bars).** *P-values indicate significant genotype×protein interactions as a predictor for remission (rs10868223, A) or response (rs11140778, B; logistic regression, age and sex as covariates).
(TIF)

Table S1 **BDNF SNPs in the MARS discovery sample.**
(DOC)

Table S2 **NTRK2 SNPs in the MARS discovery sample.**
(DOC)

Table S3 **Effect of SSRI on SNP association.**
(DOC)

Table S4 **Association with response depending on specific medication.**
(DOC)

Table S5 **Effect of gender on SNP association.**
(DOC)

Table S6 **Haplotypes Associated with Response after 5 Weeks.**
(DOC)

Document S1 **Medication- and gender-specific association.**
(DOC)

## References

[1] Hennings JM, Owashi T, Binder EB, Horstmann S, Menke A, et al (2009) Clinical characteristics and treatment outcome in a representative sample of depressed inpatients - findings from the Munich Antidepressant Response Signature (MARS) project. J Psychiatr Res 43: 215–229. 18586274 10.1016/j.jpsychires.2008.05.002

[2] Rush AJ, Trivedi MH, Wisniewski SR, Nierenberg AA, Stewart JW, et al (2006) Acute and longer-term outcomes in depressed outpatients requiring one or several treatment steps: a STAR*D report. Am J Psychiatry 163: 1905–1917. 17074942 10.1176/ajp.2006.163.11.1905

[3] Holsboer F (2000) The corticosteroid receptor hypothesis of depression. Neuropsychopharmacology 23: 477–501. 11027914 10.1016/S0893-133X(00)00159-7

[4] Duman RS, Monteggia LM (2006) A neurotrophic model for stress-related mood disorders. Biol Psychiatry 59: 1116–1127. 16631126 10.1016/j.biopsych.2006.02.013

[5] Groves JO (2007) Is it time to reassess the BDNF hypothesis of depression? Mol Psychiatry 12: 1079–1088. 17700574 10.1038/sj.mp.4002075

[6] Schaaf MJ, de Jong J, de Kloet ER, Vreugdenhil E (1998) Downregulation of BDNF mRNA and protein in the rat hippocampus by corticosterone. Brain Res 813: 112–120. 9824681 10.1016/s0006-8993(98)01010-5

[7] Smith MA, Makino S, Kvetnansky R, Post RM (1995) Stress and glucocorticoids affect the expression of brain-derived neurotrophic factor and neurotrophin-3 mRNAs in the hippocampus. J Neurosci 15: 1768–1777. 7891134 10.1523/JNEUROSCI.15-03-01768.1995 PMC6578156

[8] Poo MM (2001) Neurotrophins as synaptic modulators. Nat Rev Neurosci 2: 24–32. 11253356 10.1038/35049004

[9] Kozisek ME, Middlemas D, Bylund DB (2008) Brain-derived neurotrophic factor and its receptor tropomyosin-related kinase B in the mechanism of action of antidepressant therapies. Pharmacol Ther 117: 30–51. 17949819 10.1016/j.pharmthera.2007.07.001

[10] Sillaber I, Panhuysen M, Henniger MSH, Ohl F, Kühne C, et al (2008) Profiling of behavioral changes and hippocampal gene expression in mice chronically treated with the SSRI paroxetine. Psychopharmacology (Berl) 200: 557–572. 18629477 10.1007/s00213-008-1232-6

[11] Dwivedi Y (2009) Brain-derived neurotrophic factor: role in depression and suicide. Neuropsychiatr Dis Treat 5: 433–449. 19721723 10.2147/ndt.s5700 PMC2732010

[12] Knapman A, Heinzmann J-M, Hellweg R, Holsboer F, Landgraf R, et al (2010) Increased stress reactivity is associated with cognitive deficits and decreased hippocampal brain-derived neurotrophic factor in a mouse model of affective disorders. J Psychiatr Res 44: 566–575. 20035953 10.1016/j.jpsychires.2009.11.014

[13] Tadić A, Wagner S, Schlicht KF, Peetz D, Borysenko L, et al (2011) The early non-increase of serum BDNF predicts failure of antidepressant treatment in patients with major depression: a pilot study. Prog Neuropsychopharmacol Biol Psychiatry 35: 415–420. 20732374 10.1016/j.pnpbp.2010.08.011

[14] Saarelainen T, Hendolin P, Lucas G, Koponen E, Sairanen M, et al (2003) Activation of the TrkB neurotrophin receptor is induced by antidepressant drugs and is required for antidepressant-induced behavioral effects. J Neurosci 23: 349–357. 12514234 10.1523/JNEUROSCI.23-01-00349.2003 PMC6742146

[15] Nibuya M, Morinobu S, Duman RS (1995) Regulation of BDNF and trkB mRNA in rat brain by chronic electroconvulsive seizure and antidepressant drug treatments. J Neurosci 15: 7539–7547. 7472505 10.1523/JNEUROSCI.15-11-07539.1995 PMC6578063

[16] Rantamäki T, Knuuttila JEA, Hokkanen M-E, Castrén E (2006) The effects of acute and long-term lithium treatments on trkB neurotrophin receptor activation in the mouse hippocampus and anterior cingulate cortex. Neuropharmacology 50: 421–427. 16300803 10.1016/j.neuropharm.2005.10.001

[17] Rantamäki T, Hendolin P, Kankaanpää A, Mijatovic J, Piepponen P, et al (2007) Pharmacologically diverse antidepressants rapidly activate brain-derived neurotrophic factor receptor TrkB and induce phospholipase-Cgamma signaling pathways in mouse brain. Neuropsychopharmacology 32: 2152–2162. 17314919 10.1038/sj.npp.1301345

[18] Kato M, Serretti A (2008) Review and meta-analysis of antidepressant pharmacogenetic findings in major depressive disorder. Mol Psychiatry. Available: http://www.ncbi.nlm.nih.gov/pubmed/18982004. Accessed 2009 Sep 15. 10.1038/mp.2008.116 18982004

[19] Zou Y-F, Wang Y, Liu P, Feng X-L, Wang B-Y, et al (2010) Association of brain-derived neurotrophic factor genetic Val66Met polymorphism with severity of depression, efficacy of fluoxetine and its side effects in Chinese major depressive patients. Neuropsychobiology 61: 71–78. 20016225 10.1159/000265132

[20] Petryshen TL, Sabeti PC, Aldinger KA, Fry B, Fan JB, et al (2010) Population genetic study of the brain-derived neurotrophic factor (BDNF) gene. Mol Psychiatry 15: 810–815. 19255578 10.1038/mp.2009.24 PMC2888876

[21] Dong C, Wong M-L, Licinio J (2009) Sequence variations of ABCB1, SLC6A2, SLC6A3, SLC6A4, CREB1, CRHR1 and NTRK2: association with major depression and antidepressant response in Mexican-Americans. Mol Psychiatry 14: 1105–1118. 19844206 10.1038/mp.2009.92 PMC2834349

[22] Licinio J, Dong C, Wong M-L (2009) Novel sequence variations in the brain-derived neurotrophic factor gene and association with major depression and antidepressant treatment response. Arch Gen Psychiatry 66: 488–497. 19414708 10.1001/archgenpsychiatry.2009.38 PMC4272010

[23] Domschke K, Lawford B, Laje G, Berger K, Young R, et al.. (2009) Brain-derived neurotrophic factor (BDNF) gene: no major impact on antidepressant treatment response. Int J Neuropsychopharmacol: 1–9. 10.1017/S1461145709000030 19236730

[24] McMahon FJ, Buervenich S, Charney D, Lipsky R, Rush AJ, et al (2006) Variation in the gene encoding the serotonin 2A receptor is associated with outcome of antidepressant treatment. Am J Hum Genet 78: 804–814. 16642436 10.1086/503820 PMC1474035

[25] Baune BT, Hohoff C, Berger K, Neumann A, Mortensen S, et al (2008) Association of the COMT val158 met variant with antidepressant treatment response in major depression. Neuropsychopharmacology 33: 924–932. 17522626 10.1038/sj.npp.1301462

[26] Binder EB, Salyakina D, Lichtner P, Wochnik GM, Ising M, et al (2004) Polymorphisms in FKBP5 are associated with increased recurrence of depressive episodes and rapid response to antidepressant treatment. Nat Genet 36: 1319–1325. 15565110 10.1038/ng1479

[27] Horstmann S, Dose T, Lucae S, Kloiber S, Menke A, et al (2009) Suppressive effect of mirtazapine on the HPA system in acutely depressed women seems to be transient and not related to antidepressant action. Psychoneuroendocrinology 34: 238–248. 18926641 10.1016/j.psyneuen.2008.09.004

[28] Ising M, Horstmann S, Kloiber S, Lucae S, Binder EB, et al (2007) Combined dexamethasone/corticotropin releasing hormone test predicts treatment response in major depression - a potential biomarker? Biol Psychiatry 62: 47–54 doi:10.1016/j.biopsych.2006.07.039 17123470 10.1016/j.biopsych.2006.07.039

[29] Ising M, Lucae S, Binder EB, Bettecken T, Uhr M, et al (2009) A Genomewide Association Study Points to Multiple Loci That Predict Antidepressant Drug Treatment Outcome in Depression. Arch Gen Psychiatry 66: 966–975. 19736353 10.1001/archgenpsychiatry.2009.95 PMC4465570

[30] Uhr M, Tontsch A, Namendorf C, Ripke S, Lucae S, et al (2008) Polymorphisms in the drug transporter gene ABCB1 predict antidepressant treatment response in depression. Neuron 57: 203–209. 18215618 10.1016/j.neuron.2007.11.017

[31] Kohli MA, Salyakina D, Pfennig A, Lucae S, Horstmann S, et al (2010) Association of genetic variants in the neurotrophic receptor-encoding gene NTRK2 and a lifetime history of suicide attempts in depressed patients. Arch Gen Psychiatry 67: 348–359. 20124106 10.1001/archgenpsychiatry.2009.201 PMC3696349

[32] Skol AD, Scott LJ, Abecasis GR, Boehnke M (2006) Joint analysis is more efficient than replication-based analysis for two-stage genome-wide association studies. Nat Genet 38: 209–213. 16415888 10.1038/ng1706

[33] Colhoun H, Mckeigue P, Smith G (2003) Problems of reporting genetic associations with complex outcomes. The Lancet 361: 865–872. 10.1016/s0140-6736(03)12715-8 12642066

[34] Westfall P, Young S (1993) Resampling-Based Multiple Testing: Examples and Methods for P-Value Adjustment. New York, NY: John Wiley & Sons.

[35] North BV, Curtis D, Sham PC (2002) A Note on the Calculation of Empirical P Values from Monte Carlo Procedures. Am J Hum Genet 71: 439–441. 12111669 10.1086/341527 PMC379178

[36] Armitage P (1955) Tests for Linear Trends in Proportions and Frequencies. Biometrics 11: 375–386.

[37] Fisher RA (1932) Statistical methods for research workers. 4th ed. London: Oliver & Boyd.

[38] Barrett JC, Fry B, Maller J, Daly MJ (2005) Haploview: analysis and visualization of LD and haplotype maps. Bioinformatics 21: 263–265. 15297300 10.1093/bioinformatics/bth457

[39] Gabriel SB, Schaffner SF, Nguyen H, Moore JM, Roy J, et al (2002) The structure of haplotype blocks in the human genome. Science 296: 2225–2229. 12029063 10.1126/science.1069424

[40] Adams JH, Wigg KG, King N, Burcescu I, Vetró A, et al (2005) Association study of neurotrophic tyrosine kinase receptor type 2 (NTRK2) and childhood-onset mood disorders. Am J Med Genet B Neuropsychiatr Genet 132B: 90–95. 15389758 10.1002/ajmg.b.30084

[41] Gratacòs M, Soria V, Urretavizcaya M, González JR, Crespo JM, et al (2008) A brain-derived neurotrophic factor (BDNF) haplotype is associated with antidepressant treatment outcome in mood disorders. Pharmacogenomics J 8: 101–112. 17505499 10.1038/sj.tpj.6500460

[42] Kocabas NA, Antonijevic I, Faghel C, Forray C, Kasper S, et al (2011) Brain-derived neurotrophic factor gene polymorphisms: influence on treatment response phenotypes of major depressive disorder. Int Clin Psychopharmacol 26: 1–10. 21188787 10.1097/yic.0b013e32833d18f8

[43] Stranger BE, Montgomery SB, Dimas AS, Parts L, Stegle O, et al (2012) Patterns of Cis Regulatory Variation in Diverse Human Populations. PLoS Genet 8: e1002639. 22532805 10.1371/journal.pgen.1002639 PMC3330104

[44] Alonso P, Gratacòs M, Menchón JM, Saiz-Ruiz J, Segalàs C, et al (2008) Extensive genotyping of the BDNF and NTRK2 genes define protective haplotypes against obsessive-compulsive disorder. Biol Psychiatry 63: 619–628. 17884018 10.1016/j.biopsych.2007.06.020

[45] Cozza A, Melissari E, Iacopetti P, Mariotti V, Tedde A, et al (2008) SNPs in neurotrophin system genes and Alzheimer’s disease in an Italian population. J Alzheimers Dis 15: 61–70. 18780967 10.3233/jad-2008-15105

[46] Perroud N, Aitchison KJ, Uher R, Smith R, Huezo-Diaz P, et al. (2009) Genetic Predictors of Increase in Suicidal Ideation During Antidepressant Treatment in the GENDEP Project. Neuropsychopharmacology. Available: http://www.ncbi.nlm.nih.gov/pubmed/19641488. Accessed 16 September 2009. 10.1038/npp.2009.81 19641488

[47] Uher R, Huezo-Diaz P, Perroud N, Smith R, Rietschel M, et al (2009) Genetic predictors of response to antidepressants in the GENDEP project. Pharmacogenomics J 9: 225–233. 19365399 10.1038/tpj.2009.12

[48] Lin E, Hong C-J, Hwang J-P, Liou Y-J, Yang C-H, et al (2009) Gene-gene interactions of the brain-derived neurotrophic-factor and neurotrophic tyrosine kinase receptor 2 genes in geriatric depression. Rejuvenation Res 12: 387–393. 20014955 10.1089/rej.2009.0871

[49] Dwivedi Y, Rizavi HS, Pandey GN (2006) Antidepressants reverse corticosterone-mediated decrease in brain-derived neurotrophic factor expression: differential regulation of specific exons by antidepressants and corticosterone. Neuroscience 139: 1017–1029. 16500030 10.1016/j.neuroscience.2005.12.058 PMC1513636

[50] Choi M-J, Kang R-H, Lim S-W, Oh K-S, Lee M-S (2006) Brain-derived neurotrophic factor gene polymorphism (Val66Met) and citalopram response in major depressive disorder. Brain Res 1118: 176–182. 16979146 10.1016/j.brainres.2006.08.012

[51] Kang R, Chang H, Wong M, Choi M, Park J, et al. (2009) Brain-derived neurotrophic factor gene polymorphisms and mirtazapine responses in Koreans with major depression. J Psychopharmacol (Oxford). Available: http://www.ncbi.nlm.nih.gov/pubmed/19493959. Accessed 2009 Sep 14. 10.1177/0269881109105457 19493959

[52] Tsai S-J, Hong C-J, Liou Y-J (2008) Brain-derived neurotrophic factor and antidepressant action: another piece of evidence from pharmacogenetics. Pharmacogenomics 9: 1353–1358. 18781861 10.2217/14622416.9.9.1353

[53] Schumacher J, Jamra RA, Becker T, Ohlraun S, Klopp N, et al (2005) Evidence for a relationship between genetic variants at the brain-derived neurotrophic factor (BDNF) locus and major depression. Biol Psychiatry 58: 307–314. 16005437 10.1016/j.biopsych.2005.04.006

[54] Taylor WD, McQuoid DR, Ashley-Koch A, Macfall JR, Bridgers J, et al. (2010) BDNF Val66Met genotype and 6-month remission rates in late-life depression. Pharmacogenomics J. Available: http://www.ncbi.nlm.nih.gov/pubmed/20195291. Accessed 2011 Jan 25. 10.1038/tpj.2010.12 PMC2962689 20195291

[55] Wilkie MJV, Smith D, Reid IC, Day RK, Matthews K, et al (2007) A splice site polymorphism in the G-protein beta subunit influences antidepressant efficacy in depression. Pharmacogenet Genomics 17: 207–215. 17460549 10.1097/FPC.0b013e32801a3be6

[56] Yoshida K, Higuchi H, Kamata M, Takahashi H, Inoue K, et al (2007) The G196A polymorphism of the brain-derived neurotrophic factor gene and the antidepressant effect of milnacipran and fluvoxamine. J Psychopharmacol (Oxford) 21: 650–656. 17092970 10.1177/0269881106072192

[57] Verhagen M, van der Meij A, van Deurzen PAM, Janzing JGE, Arias-Vásquez A, et al (2010) Meta-analysis of the BDNF Val66Met polymorphism in major depressive disorder: effects of gender and ethnicity. Mol Psychiatry 15: 260–271. 18852698 10.1038/mp.2008.109

[58] Hong C-J, Huo S-J, Yen F-C, Tung C-L, Pan G-M, et al (2003) Association study of a brain-derived neurotrophic-factor genetic polymorphism and mood disorders, age of onset and suicidal behavior. Neuropsychobiology 48: 186–189. 14673216 10.1159/000074636

[59] Kunugi H, Iijima Y, Tatsumi M, Yoshida M, Hashimoto R, et al (2004) No association between the Val66Met polymorphism of the brain-derived neurotrophic factor gene and bipolar disorder in a Japanese population: a multicenter study. Biol Psychiatry 56: 376–378. 15336520 10.1016/j.biopsych.2004.06.017

[60] Nakata K, Ujike H, Sakai A, Uchida N, Nomura A, et al (2003) Association study of the brain-derived neurotrophic factor (BDNF) gene with bipolar disorder. Neurosci Lett 337: 17–20. 12524161 10.1016/s0304-3940(02)01292-2

[61] Neves-Pereira M, Mundo E, Muglia P, King N, Macciardi F, et al (2002) The brain-derived neurotrophic factor gene confers susceptibility to bipolar disorder: evidence from a family-based association study. Am J Hum Genet 71: 651–655. 12161822 10.1086/342288 PMC379201

[62] Ghaemi SN, Ko JY, Goodwin FK (2001) The bipolar spectrum and the antidepressant view of the world. J Psychiatr Pract 7: 287–297. 15990539 10.1097/00131746-200109000-00002

[63] Mitchell PB, Goodwin GM, Johnson GF, Hirschfeld RMA (2008) Diagnostic guidelines for bipolar depression: a probabilistic approach. Bipolar Disord 10: 144–152. 18199233 10.1111/j.1399-5618.2007.00559.x

[64] Angst J, Sellaro R, Stassen HH, Gamma A (2005) Diagnostic conversion from depression to bipolar disorders: results of a long-term prospective study of hospital admissions. J Affect Disord 84: 149–157. 15708412 10.1016/S0165-0327(03)00195-2

[65] McMahon FJ, Akula N, Schulze TG, Muglia P, Tozzi F, et al (2010) Meta-analysis of genome-wide association data identifies a risk locus for major mood disorders on 3p21.1. Nat Genet 42: 128–131. 20081856 10.1038/ng.523 PMC2854040

[66] Baghai TC, Binder EB, Schule C, Salyakina D, Eser D, et al (2006) Polymorphisms in the angiotensin-converting enzyme gene are associated with unipolar depression, ACE activity and hypercortisolism. Mol Psychiatry 11: 1003–1015. 16924268 10.1038/sj.mp.4001884

[67] Wittke-Thompson JK, Pluzhnikov A, Cox NJ (2005) Rational inferences about departures from Hardy-Weinberg equilibrium. Am J Hum Genet 76: 967–986. 15834813 10.1086/430507 PMC1196455

