# Supplementary figures and images for: Possible Associations of NTRK2 Polymorphisms with Antidepressant Treatment Outcome: Findings from an Extended Tag SNP Approach
Source: PLoS One. 2013 Jun 4;8(6):e64947. doi: 10.1371/journal.pone.0064947 (PMC3672143; doi:10.1371/journal.pone.0064947)

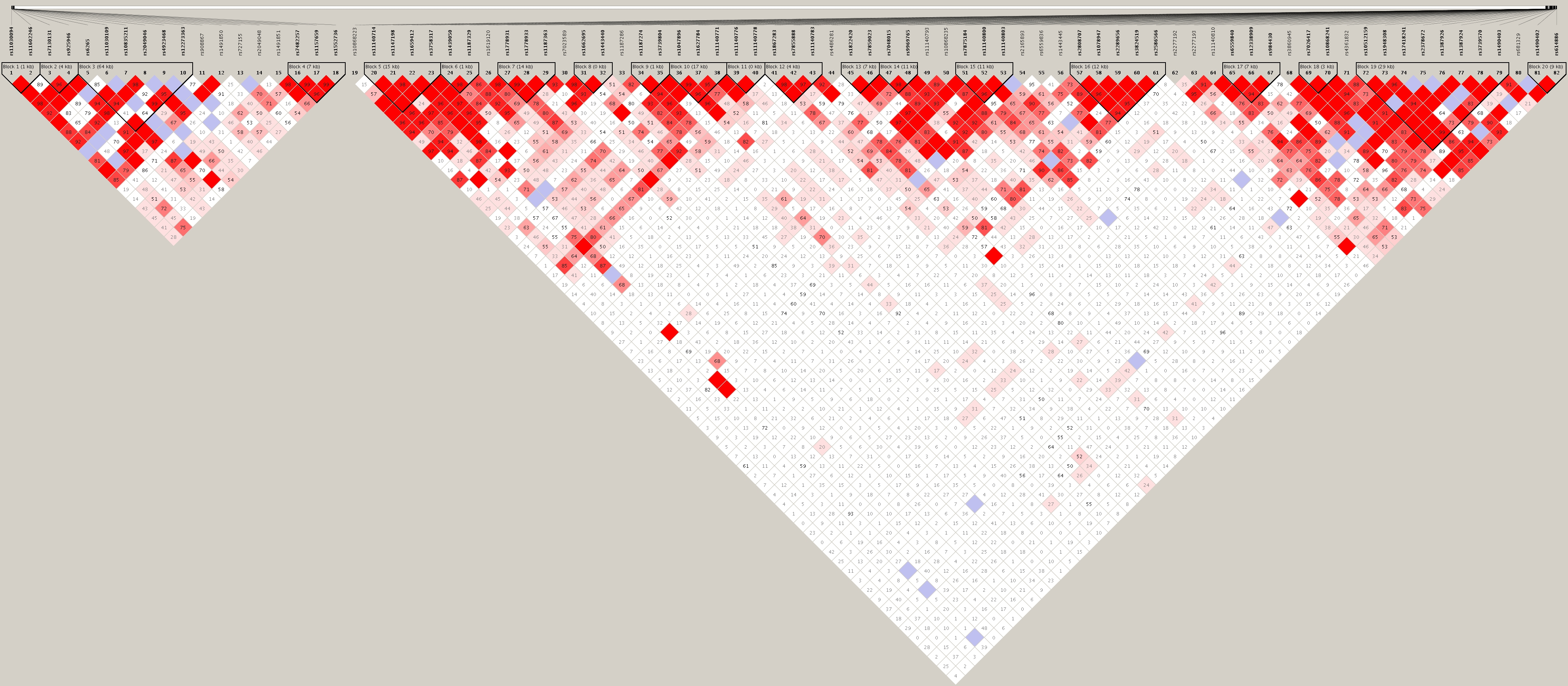

Supplement: Figure S1 — D’-based linkage disequilibrium (LD) of the BDNF (left panel) and NTRK2 (right panel) gene regions retrieved from HapMap for the CEU population (release 21). Haplotype blocks were defined according to Gabriel et al. [39]. (TIF) [file pone.0064947.s001.tif]

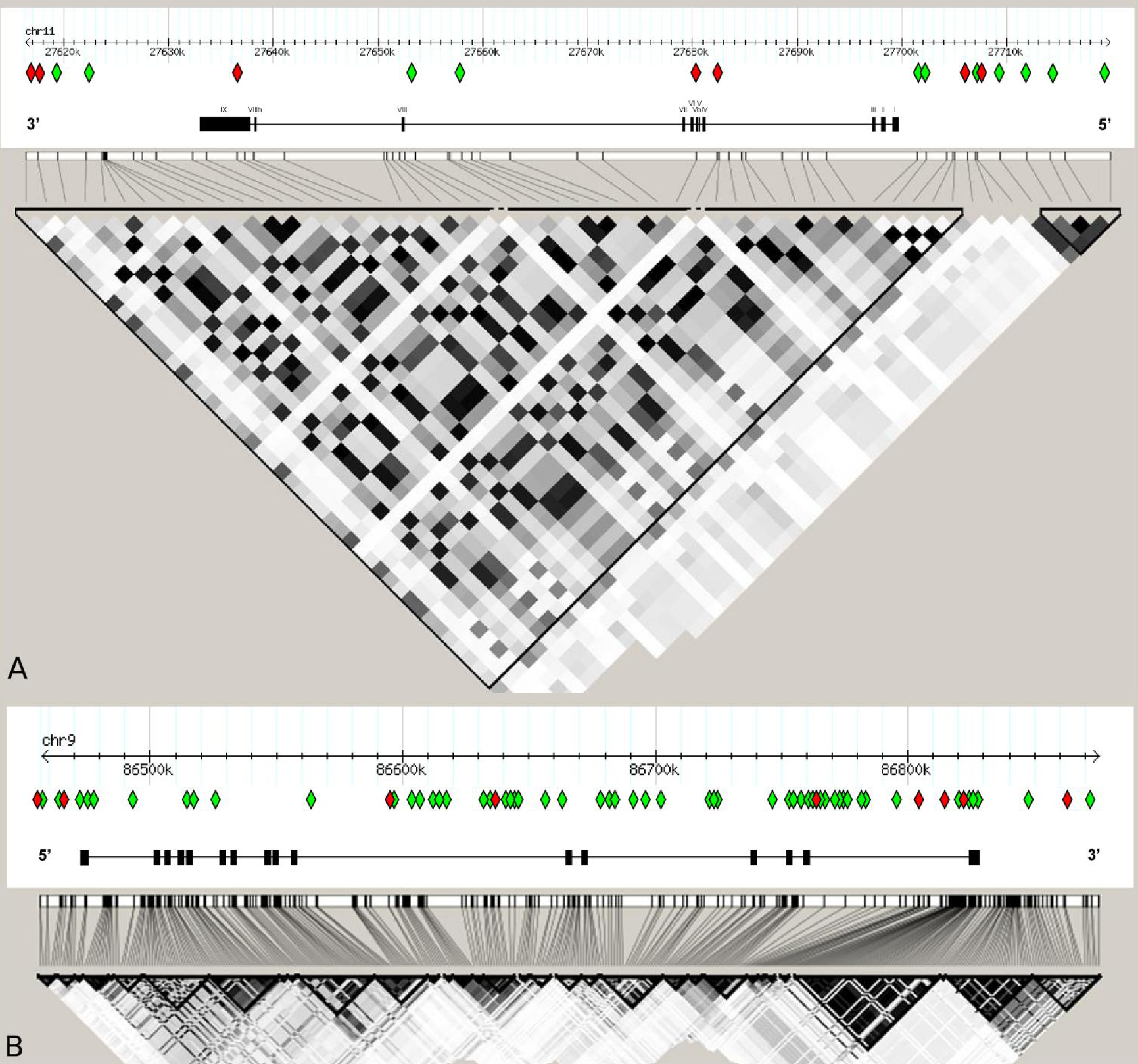

Supplement: Figure S2 — R2-based linkage disequilibrium (LD) of the BDNF (A) and NTRK2 (B) gene regions retrieved from HapMap for the CEU population (release 21). Diamonds indicate genotyped SNPs in the discovery sample. Red diamonds represent nominally associated SNPs in the discovery sample. The exon (black boxes)-intron structures of the longest isoforms of BDNF (NM_170731) and NTRK2 (NM_006180) are depicted according to dbSNP build 132. In case of BDNF, black boxes indicate alternative exons (I-IX) as proposed by Pruunsild, et al (Pruunsild, et al 2007), and are approximated relative to the SNPs. (TIFF) [file pone.0064947.s002.tiff]

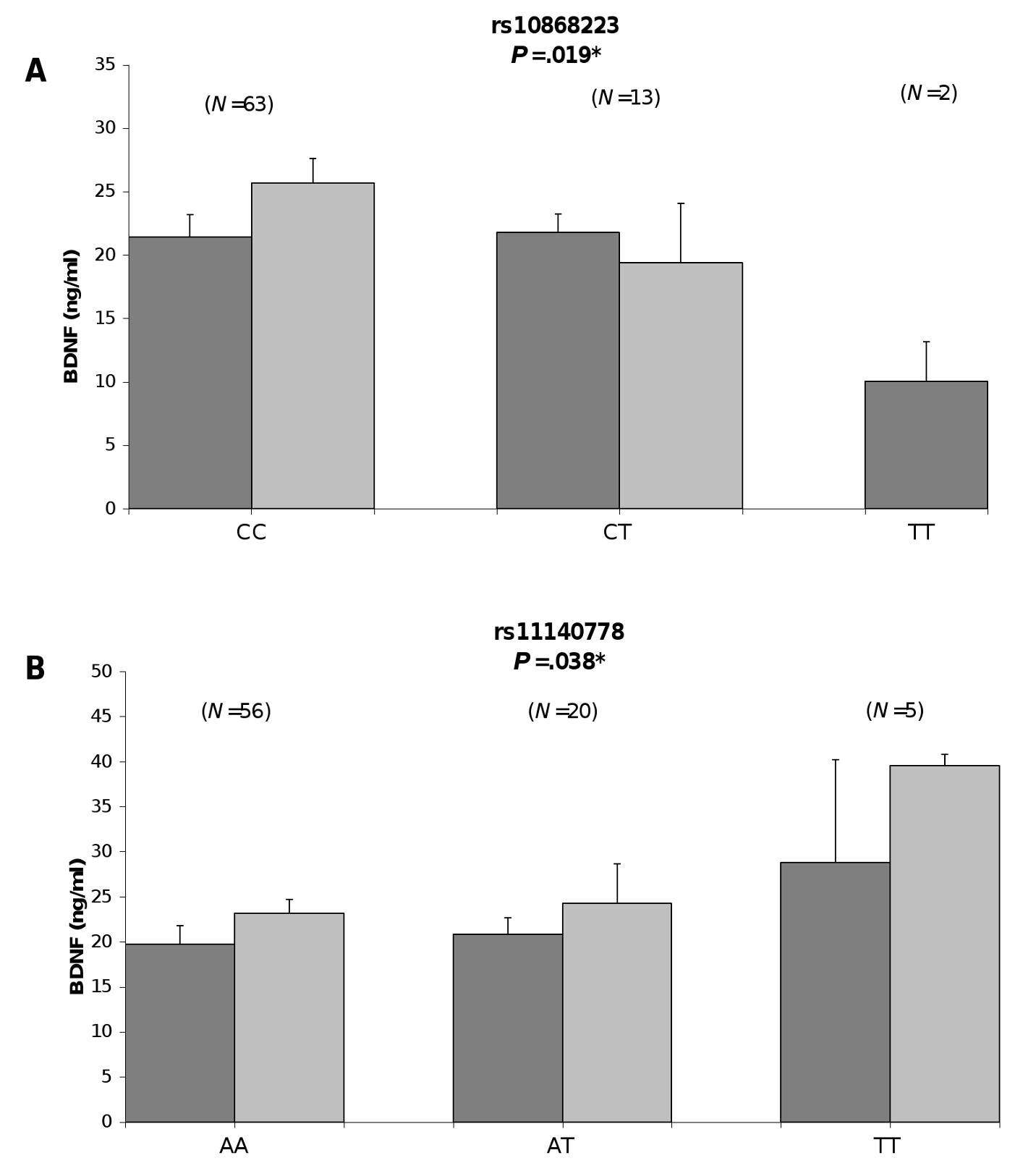

Supplement: Figure S3 — Genotype-dependent concentration of serum BDNF levels in non-improved (dark grey bars) and improved patients (light grey bars). *P-values indicate significant genotype×protein interactions as a predictor for remission (rs10868223, A) or response (rs11140778, B; logistic regression, age and sex as covariates). (TIF) [file pone.0064947.s003.tif]
