# Supplementary material for: Possible Associations of NTRK2 Polymorphisms with Antidepressant Treatment Outcome: Findings from an Extended Tag SNP Approach
Source: PLoS One. 2013 Jun 4;8(6):e64947. doi: 10.1371/journal.pone.0064947 (PMC3672143; doi:10.1371/journal.pone.0064947)
Supplement: Table S1 — BDNF SNPs in the MARS discovery sample. (DOC) [file pone.0064947.s004.doc]

| **Table S1. *BDNF* SNPs in the MARS discovery sample** | | | | | | |  |  |  |
| --- | --- | --- | --- | --- | --- | --- | --- | --- | --- |
|  |  |  |  |  |  | **Response after 5 weeks** | | **Remission at discharge** | |
| **SNP** | **Map position^a^** | **Function^b^** | **Minor/ major allele** | **MAF** | **HWE *P*^c^** | ***P*^d^ (allelic)** | ***P*^d^ (genotypic)** | ***P*^d^ (allelic)** | ***P*^d^ (genotypic)** |
| rs1552736 | 27719853 | 5' | A/G | .36 | .38 | .97 | .48 | .62 | .47 |
| rs1157659 | 27714198 | 5' | A/G | .48 | .76 | .19 | .42 | .92 | .34 |
| rs7482257 | 27711874 | 5' | T/C | .30 | .28 | .84 | .94 | .63 | .06 |
| rs1491851 | 27709339 | 5' | T/C | .41 | .92 | .22 | .42 | .65 | .12 |
| rs2049048 | 27707162 | 5' | T/C | .17 | >.99 | **.014** | **.008** | .47 | .38 |
| rs727155 | 27707025 | 5' | T/C | .04 | >.99 | .45 | >.99 | .58 | >.99 |
| rs1491850 | 27706301 | 5' | C/T | .46 | .42 | **.009** | **.031** | **.003** | **.01** |
| rs908867 | 27702340 | 5' | A/G | .09 | .19 | .13 | .32 | .13 | .13 |
| rs12273363 | 27701435 | 5' | C/T | .18 | .39 | .42 | .49 | **.02** | **.049** |
| rs4923468 | 27682351 | intron | A/C | .01 | >.99 | **.01** | >.99 | .54 | >.99 |
| rs2049046 | 27680351 | intron | A/T | .45 | .03 | **4.88x10^-5^** | **4.66x10^-4^** | **1.09x10^-4^** | **5.70x10^-4^** |
| rs10835211 | 27657940 | intron | A/G | .22 | .19 | .40 | .32 | **.005** | **.02** |
| rs11030109 | 27653527 | intron | A/G | .02 | >.99 | .58 | >.99 | .95 | >.99 |
| rs6265 | 27636491 | Val66Met | A/G | .23 | .04 | **.04** | .07 | .31 | .62 |
| rs925946 | 27623778 | 3' | T/G | .27 | .01 | .16 | .32 | **.004** | **.01** |
| rs7130131 | 27619402 | 3' | C/T | .06 | .36 | .16 | .32 | .45 | .22 |
| rs11602246 | 27617502 | 3' | G/C | .10 | .17 | **.009** | **.034** | .06 | .14 |
| rs11030094 | 27616351 | 3' | A/G | .42 | .10 | **1.52x10^-4^** | **6.05x10^-4^** | **.001** | **.002** |
| ^a^ Position on chromosome 11, according to hg18 | | | | | | | |  |  |
| ^b^ According to dbSNP build 132 | | | | | | | |  |  |
| ^c^ Uncorrected *P* values from the discovery sample for the deviation from Hardy-Weinberg-Equilibrium; note that no *P* value exceeded the corrected (82 SNPs, Bonferroni) threshold of p<6.1x10^-4^. | | | | | | | |  |  |
| ^d^ Nominal *P* values | | | | | | | |  |  |
