# Supplementary material for: Possible Associations of NTRK2 Polymorphisms with Antidepressant Treatment Outcome: Findings from an Extended Tag SNP Approach
Source: PLoS One. 2013 Jun 4;8(6):e64947. doi: 10.1371/journal.pone.0064947 (PMC3672143; doi:10.1371/journal.pone.0064947)
Supplement: Table S2 — NTRK2 SNPs in the MARS discovery sample. (DOC) [file pone.0064947.s005.doc]

| **Table S2. *NTRK2* SNPs in the MARS discovery sample** | | | | | | |  |  |  |
| --- | --- | --- | --- | --- | --- | --- | --- | --- | --- |
|  |  |  |  |  |  | **Response after 5 weeks** | | **Remission at discharge** | |
| **SNP** | **Map position^a^** | **Function^b^** | **Minor/ major allele^c^** | **MAF** | **HWE *P*^c^** | ***P*^d^ (allelic)** | ***P*^d^ (genotypic)** | ***P*^d^ (allelic)** | ***P*^d^ (genotypic)** |
| rs10868223 | 86454945 | 5' | T/C | .12 | .35 | **.03** | .11 | .11 | .28 |
| rs11140714 | 86456519 | 5' | G/A | .24 | .69 | .43 | .64 | .88 | .85 |
| rs1147198 | 86465167 | 5' | C/A | .22 | >.99 | .12 | .21 | .99 | .91 |
| rs1659412 | 86467971 | 5' | C/T | .10 | .08 | **.02** | .08 | .17 | .36 |
| rs3758317 | 86472434 | 5' | T/C | .20 | .17 | .90 | .99 | .75 | .92 |
| rs1439050 | 86478012 | intron | T/G | .35 | .51 | .42 | .42 | .61 | .07 |
| rs1187329 | 86479099 | intron | A/G | .45 | .84 | .51 | .51 | .82 | .09 |
| rs1619120 | 86492015 | intron | T/C | .42 | .47 | .37 | .64 | .43 | .41 |
| rs1778931 | 86513594 | intron | A/G | .49 | .84 | .56 | .83 | .29 | .11 |
| rs1778933 | 86514230 | intron | C/T | .36 | .52 | .24 | .46 | .21 | .41 |
| rs1187363 | 86527739 | intron | A/G | .19 | .34 | .29 | .58 | .52 | .59 |
| rs7023589 | 86561916 | intron | G/A | .35 | .74 | .25 | .44 | .87 | .94 |
| rs1662695 | 86595031 | intron | C/T | .13 | .37 | **.04** | .11 | .35 | .63 |
| rs1443440 | 86595987 | intron | G/A | .29 | .81 | .19 | .38 | .34 | .58 |
| rs1187286 | 86604847 | intron | C/A | .24 | .34 | .33 | .62 | .11 | .28 |
| rs1187274 | 86609608 | intron | G/C | .30 | >.99 | .61 | .86 | .16 | .10 |
| rs3739804 | 86611450 | intron | G/A | .08 | .49 | .22 | .48 | .27 | .41 |
| rs1047896 | 86615792 | intron | C/T | .19 | .51 | .30 | .53 | .29 | .44 |
| rs1627784 | 86618685 | intron | G/A | .27 | .44 | .87 | .80 | .15 | .04 |
| rs11140771 | 86633181 | intron | A/G | .20 | .88 | .50 | .47 | .22 | .33 |
| rs11140776 | 86636746 | intron | T/G | .46 | .92 | .13 | .07 | .78 | .91 |
| rs11140778 | 86636977 | intron | T/A | .21 | >.99 | **.004** | **.014** | .22 | .48 |
| rs1867283 | 86640585 | intron | A/G | .49 | >.99 | .66 | .45 | .90 | .93 |
| rs7855888 | 86641877 | intron | C/T | .25 | .69 | .77 | .90 | .98 | .98 |
| rs11140783 | 86644636 | intron | T/C | .09 | .76 | .69 | .36 | .73 | .46 |
| rs4486281 | 86645166 | intron | G/A | .29 | .39 | .32 | .08 | .50 | .80 |
| rs1822420 | 86657095 | intron | T/C | .14 | .14 | .55 | .80 | .30 | .30 |
| rs7859023 | 86664239 | intron | G/A | .15 | .12 | .29 | .54 | .61 | .41 |

| **Table S2. *NTRK2* SNPs in the MARS discovery sample (cont)** | | | | | | | |  |  |
| --- | --- | --- | --- | --- | --- | --- | --- | --- | --- |
|  |  |  |  |  |  | **Response after 5 weeks** | | **Remission at discharge** | |
| **SNP** | **Map position^a^** | **Function^b^** | **Minor/ major allele^c^** | **MAF** | **HWE *P*^c^** | ***P*^d^ (allelic)** | ***P*^d^ (genotypic)** | ***P*^d^ (allelic)** | ***P*^d^ (genotypic)** |
| rs7048015 | 86667954 | intron | C/A | .18 | .09 | .53 | .83 | **.01** | **.04** |
| rs9969765 | 86679604 | intron | G/C | .33 | .02 | .33 | .59 | **.03** | .09 |
| rs11140793 | 86681299 | intron | C/A | .15 | .04 | .49 | .80 | .06 | .13 |
| rs10868235 | 86683574 | intron | C/T | .46 | >.99 | .24 | .51 | .27 | .54 |
| rs7875184 | 86690917 | intron | T/C | .14 | .83 | .81 | .30 | .96 | .54 |
| rs11140800 | 86697956 | intron | C/A | .41 | .22 | .82 | .62 | .48 | .76 |
| rs11140803 | 86702488 | intron | A/G | .17 | .72 | .66 | .73 | .50 | .29 |
| rs2165893 | 86721243 | intron | G/A | .06 | .67 | .23 | .29 | .35 | 1.00 |
| rs6559836 | 86723208 | intron | G/A | .17 | .86 | .80 | .45 | .38 | .66 |
| rs1443445 | 86723977 | intron | C/T | .20 | .12 | .22 | .24 | .85 | .40 |
| rs2808707 | 86748113 | intron | T/G | .40 | >.99 | .87 | .99 | .99 | >.99 |
| rs1078947 | 86753071 | intron | A/G | .15 | .56 | .28 | .44 | .81 | .17 |
| rs2289656 | 86753381 | intron | T/C | .17 | .48 | .38 | .08 | .50 | .72 |
| rs3824519 | 86759823 | intron | A/G | .08 | >.99 | .88 | .29 | .26 | .39 |
| rs2586566 | 86760731 | intron | G/A | .23 | .15 | .69 | .74 | .90 | .38 |
| rs2277192 | 86763796 | intron | G/A | .15 | .85 | .50 | .57 | .61 | .73 |
| rs2277193 | 86763828 | intron | C/T | .28 | .46 | **.02** | .08 | .96 | .30 |
| rs11140810 | 86764627 | intron | T/G | .44 | .54 | .06 | **.02** | .67 | .12 |
| rs6559840 | 86765319 | intron | T/C | .27 | .44 | .13 | .22 | .71 | .27 |
| rs12338909 | 86770878 | intron | T/G | .10 | >.99 | .65 | .88 | .93 | .98 |
| rs984430 | 86772646 | intron | A/G | .17 | .29 | .40 | .68 | .16 | .30 |
| rs3860945 | 86775443 | intron | G/A | .14 | .20 | .12 | .12 | .10 | .14 |
| rs7026417 | 86778984 | intron | C/T | .10 | .16 | .70 | .91 | .50 | .66 |
| rs10868241 | 86782847 | intron | A/G | .28 | .80 | .45 | .62 | .45 | .18 |
| rs4361832 | 86785553 | intron | A/G | .18 | .61 | .31 | .47 | .79 | .95 |
| rs10512159 | 86798818 | intron | A/G | .16 | .85 | .31 | .33 | .31 | .51 |
| rs1948308 | 86806076 | intron | C/T | .44 | .61 | **.008** | **.008** | .07 | .12 |
| rs17418241 | 86815667 | intron | T/C | .09 | .75 | **.04** | .09 | .07 | .16 |

| **Table S2. *NTRK2* SNPs in the MARS discovery sample (cont)** | | | | | | | |  |  |
| --- | --- | --- | --- | --- | --- | --- | --- | --- | --- |
|  |  |  |  |  |  | **Response after 5 weeks** | | **Remission at discharge** | |
| **SNP** | **Map position^a^** | **Function^b^** | **Minor/ major allele^c^** | **MAF** | **HWE *P*^c^** | ***P*^d^ (allelic)** | ***P*^d^ (genotypic)** | ***P*^d^ (allelic)** | ***P*^d^ (genotypic)** |
| rs2378672 | 86820853 | intron | G/A | .08 | >.99 | .44 | .71 | .81 | .13 |
| rs1387926 | 86822482 | intron | A/G | .12 | .17 | **.047** | .08 | .05 | .10 |
| rs1387924 | 86822812 | intron | A/C | .17 | .48 | .09 | .12 | .85 | .32 |
| rs3739570 | 86827397 | 3' UTR | T/C | .10 | .15 | .53 | .55 | .71 | >.99 |
| rs1490403 | 86828325 | 3' | A/T | .28 | .22 | .40 | .45 | .92 | .90 |
| rs681329 | 86849218 | 3' | C/T | .12 | .49 | .72 | .43 | .47 | .57 |
| rs1490402 | 86863072 | 3' | G/A | .14 | .40 | **.009** | **.015** | **.02** | **.04** |
| rs614886 | 86872294 | 3' | T/G | .30 | .23 | .18 | .35 | .47 | .69 |
| ^a^ Position on chromosome 9, according to hg18 | | | | | | | |  |  |
| ^b^ According to dbSNP build 132 | | | | | | | |  |  |
| ^c^ Uncorrected *P* values from the discovery sample for the deviation from Hardy-Weinberg-Equilibrium; note that no *P* value exceeded the corrected (82 SNPs, Bonferroni) threshold of p<6.1x10^-4^. | | | | | | | | | |
| ^d^ Nominal *P* values | | | | | | | |  |  |
