# Supplementary material for: Possible Associations of NTRK2 Polymorphisms with Antidepressant Treatment Outcome: Findings from an Extended Tag SNP Approach
Source: PLoS One. 2013 Jun 4;8(6):e64947. doi: 10.1371/journal.pone.0064947 (PMC3672143; doi:10.1371/journal.pone.0064947)
Supplement: Table S3 — Effect of SSRI on SNP association. (DOC) [file pone.0064947.s006.doc]

| **Table S3. Effect of SSRI on SNP association** | | | | | | | |
| --- | --- | --- | --- | --- | --- | --- | --- |
|  |  |  | **with SSRI** | |  | **without SSRI** | |
|  |  |  | ***N*=281** | |  | ***N*=589** | |
| **SNP** | **Gene** |  | ***P^a^*** | ***P^b^*** |  | ***P^a^*** | ***P^b^*** |
| rs2049048 | *BDNF* |  | .34 | .32 |  | .04 | .42 |
| rs1491850 | *BDNF* |  | .67 | .55 |  | **.05** | .49 |
| rs4923468 | *BDNF* |  | .33 | .31 |  | .23 | .98 |
| rs2049046 | *BDNF* |  | **.001** | **.004** |  | .16 | .88 |
| rs6265 | *BDNF* |  | .25 | .24 |  | .13 | .85 |
| rs11602246 | *BDNF* |  | .725 | .61 |  | .21 | .94 |
| rs11030094 | *BDNF* |  | **.049** | .08 |  | .11 | .78 |
| rs10868223 | *NTRK2* |  | .18 | .20 |  | **.002** | **.02** |
| rs1659412 | *NTRK2* |  | **.017** | .04 |  | **.02** | .21 |
| rs1662695 | *NTRK2* |  | **.02** | .04 |  | .37 | >.99 |
| rs11140778 | *NTRK2* |  | **.001** | **.007** |  | **.03** | .43 |
| rs2277193 | *NTRK2* |  | .36 | .35 |  | .92 | >.99 |
| rs1948308 | *NTRK2* |  | .06 | .10 |  | .15 | .91 |
| rs17418241 | *NTRK2* |  | .59 | .51 |  | .31 | .99 |
| rs1387926 | *NTRK2* |  | .50 | .45 |  | .51 | >.99 |
| rs1490402 | *NTRK2* |  | .36 | .35 |  | .19 | .94 |
| ^a^ Empirical *P* values for the associations with treatment outcome (FPM analysis) under an allelic model | | | | | | | |
| ^b^ Permutation-based corrected *P* value (16 SNPs) | | | | | | | |
