# Supplementary material for: Possible Associations of NTRK2 Polymorphisms with Antidepressant Treatment Outcome: Findings from an Extended Tag SNP Approach
Source: PLoS One. 2013 Jun 4;8(6):e64947. doi: 10.1371/journal.pone.0064947 (PMC3672143; doi:10.1371/journal.pone.0064947)
Supplement: Table S4 — Association with response depending on specific medication. (DOC) [file pone.0064947.s007.doc]

| **Table S4. Association with response depending on specific medication** | | | | | | | | | | |
| --- | --- | --- | --- | --- | --- | --- | --- | --- | --- | --- |
|  |  |  | **TCA only** | |  | **SNRI or NASSA only** | |  | **SSRI only** | |
|  |  |  | ***N*=162** | |  | ***N*=253** | |  | ***N*=215** | |
| **SNP^a^** | **Gene** |  | ***P^a^*** | ***P^b^*** |  | ***P^a^*** | ***P^b^*** |  | ***P^a^*** | ***P^b^*** |
| rs2049048 | *BDNF* |  | .28 | .99 |  | **.12** | .79 |  | .56 | >.99 |
| rs1491850 | *BDNF* |  | .50 | >.99 |  | .19 | .94 |  | .78 | >.99 |
| rs4923468 | *BDNF* |  | .32 | .97 |  | .12 | .86 |  | .17 | .94 |
| rs2049046 | *BDNF* |  | .39 | >.99 |  | .46 | >.99 |  | **.002** | **.02** |
| rs6265 | *BDNF* |  | .30 | .99 |  | .11 | .73 |  | .47 | >.99 |
| rs11602246 | *BDNF* |  | .14 | .83 |  | .03 | .22 |  | .57 | >.99 |
| rs11030094 | *BDNF* |  | .29 | >.99 |  | .14 | .88 |  | **.11** | .76 |
| rs10868223 | *NTRK2* |  | .41 | >.99 |  | .14 | .86 |  | .37 | >.99 |
| rs1659412 | *NTRK2* |  | .26 | .99 |  | .03 | .34 |  | **.02** | .24 |
| rs1662695 | *NTRK2* |  | .16 | .92 |  | **.05** | .63 |  | **.01** | .06 |
| rs11140778 | *NTRK2* |  | .39 | >.99 |  | .23 | .98 |  | **.002** | **.04** |
| rs2277193 | *NTRK2* |  | .71 | >.99 |  | .83 | >.99 |  | .12 | .87 |
| rs1948308 | *NTRK2* |  | .93 | >.99 |  | **.26** | .99 |  | .17 | .95 |
| rs17418241 | *NTRK2* |  | .13 | .77 |  | .92 | >.99 |  | .67 | >.99 |
| rs1387926 | *NTRK2* |  | .24 | .97 |  | .48 | >.99 |  | .95 | >.99 |
| rs1490402 | *NTRK2* |  | .08 | .62 |  | .74 | >.99 |  | .73 | >.99 |
| ^a^ Empirical *P* values for the associations with treatment outcome (FPM analysis) under an allelic model | | | | | | | | | | |
| ^b^ Permutation-based corrected *P* value (16 SNPs) | | | | | | | | | | |
