# Supplementary material for: Possible Associations of NTRK2 Polymorphisms with Antidepressant Treatment Outcome: Findings from an Extended Tag SNP Approach
Source: PLoS One. 2013 Jun 4;8(6):e64947. doi: 10.1371/journal.pone.0064947 (PMC3672143; doi:10.1371/journal.pone.0064947)
Supplement: Table S5 — Effect of gender on SNP association. (DOC) [file pone.0064947.s008.doc]

| **Table S5. Effect of gender on SNP association** | | | | | | |  |
| --- | --- | --- | --- | --- | --- | --- | --- |
|  |  |  | **Males** | |  | **Females** | |
|  |  |  | ***N*=397** | |  | ***N*=497** | |
| **SNP** | **Gene** |  | ***P^a^*** | ***P^b^*** |  | ***P^a^*** | ***P^b^*** |
| rs2049048 | *BDNF* |  | .07 | .54 |  | **.01** | .23 |
| rs1491850 | *BDNF* |  | .19 | .94 |  | .21 | .96 |
| rs4923468 | *BDNF* |  | .58 | >.99 |  | .21 | .96 |
| rs2049046 | *BDNF* |  | **.02** | .17 |  | .05 | .51 |
| rs6265 | *BDNF* |  | .29 | .99 |  | .20 | .93 |
| rs11602246 | *BDNF* |  | .29 | .98 |  | .72 | >.99 |
| rs11030094 | *BDNF* |  | .08 | .63 |  | .10 | .77 |
| rs10868223 | *NTRK2* |  | **.01** | .09 |  | .07 | .64 |
| rs1659412 | *NTRK2* |  | .26 | .98 |  | **.001** | **.02** |
| rs1662695 | *NTRK2* |  | .33 | >.99 |  | **.04** | .38 |
| rs11140778 | *NTRK2* |  | **3.5x10^-4^** | **.01** |  | .05 | .57 |
| rs2277193 | *NTRK2* |  | .82 | >.99 |  | .48 | >.99 |
| rs1948308 | *NTRK2* |  | .27 | .99 |  | **.02** | .24 |
| rs17418241 | *NTRK2* |  | .79 | >.99 |  | .18 | .93 |
| rs1387926 | *NTRK2* |  | .70 | >.99 |  | **.04** | .47 |
| rs1490402 | *NTRK2* |  | .93 | >.99 |  | **.01** | .15 |
| ^a^ Empirical *P* valuesfor the associations with treatment outcome (FPM analysis) under an allelic model | | | | | | | |
| ^b^ Permutation-based corrected *P* value (16 SNPs) | | | | | | | |
