# Supplementary material for: Possible Associations of NTRK2 Polymorphisms with Antidepressant Treatment Outcome: Findings from an Extended Tag SNP Approach
Source: PLoS One. 2013 Jun 4;8(6):e64947. doi: 10.1371/journal.pone.0064947 (PMC3672143; doi:10.1371/journal.pone.0064947)
Supplement: Table S6 — Haplotypes Associated with Response after 5 Weeks. (DOC) [file pone.0064947.s009.doc]

| **Table S6. Haplotypes Associated with Response after 5 Weeks** | | | | | |  |
| --- | --- | --- | --- | --- | --- | --- |
| **Block** | **Gene** | **Haplotype^a^** | **Frequency (Responder, Non-responder)** | ***χ^2^*** | ***P^b^*** | ***P^c^*** |
| 1 | *BDNF* | GC | 0.641, 0.505 | *14.348* | **2.0x10^-4^** | **.007** |
|  |  | AC | 0.281, 0.359 | 5.272 | .02 | .65 |
|  |  | AG | 0.078, 0.136 | 6.739 | .009 | .35 |
| 3 | *BDNF* | GGGACT | 0.356, 0.492 | 14.375 | **1.0x10^-4^** | **.007** |
|  |  | AGGTCT | 0.260, 0.196 | 4.463 | .03 | .81 |
|  |  | GGGTCT | 0.115, 0.074 | 3.826 | .05 | .90 |
|  |  | GGGTAT | 0.021, 0.003 | 5.472 | .02 | .61 |
| 5 | *NTRK2* | ACCC | 0.128, 0.074 | 6.142 | .01 | .45 |
| 6 | *NTRK2* | GA | 0.136, 0.079 | 6.449 | .01 | .40 |
| 8 | *NTRK2* | CG | 0.151, 0.102 | 4.148 | .04 | .85 |
| 11 | *NTRK2* | TT | 0.169, 0.254 | 8.23 | .004 | .18 |
| 17 | *NTRK2* | CGG | 0.530, 0.602 | 4.045 | .04 | .87 |
| 19 | *NTRK2* | GTCAGCCT | 0.375, 0.445 | 3.88 | .05 | .89 |
| 20 | *NTRK2* | GT | 0.167, 0.102 | 6.861 | .009 | .33 |
| ^a^ Only nominal significant haplotypes are reported. Haplotype analysis was performed in the discovery sample providing sufficient gene coverage (82 SNPs). Underlined letters indicate SNPs that have shown also nominal significant association in the single marker analysis | | | | | | |
| ^b^ Nominal *P* value from a χ2 analysis implemented in Haploview. | | | | | | |
| ^c^ Permutation-based corrected *P* values | | | | | | |
