# Supplementary material for: Possible Associations of NTRK2 Polymorphisms with Antidepressant Treatment Outcome: Findings from an Extended Tag SNP Approach
Source: PLoS One. 2013 Jun 4;8(6):e64947. doi: 10.1371/journal.pone.0064947 (PMC3672143; doi:10.1371/journal.pone.0064947)
Supplement: Document S1 — Medication- and gender-specific association. (DOC) [file pone.0064947.s010.doc]

**Document S1: Medication- and gender-specific association**

We tested, whether differences in response within the three genotypes of rs10868223, rs1659412 and rs11140778 were related to the type of prescribed antidepressants. We found significant genotype-dependent differences (at discharge) only for rs1659412 (without/with SSRI: CC, 6; CT, 95; TT, 485 *vs.* CC, 6; CT, 65; TT, 206; *Χ^2^* =8.75, *df*=2, *P*=.013, *N*=863). No further differences in medication were found for rs1659412 (TCA 18.8%, *P*=.65; SNRI

SNRS or NASSA 48.8%, *P*=.15; NARI 7.8%, *P*=.80; MAOI and others 13.1%, *P*=.86) nor for rs10868223 (TCA 19.1%, *P*=.65; SNRI

SNRS or NASSA 48.5%, *P*=.34; SSRI 32.5%, *P*=.81; NARI 7.8%, *P*=.28; MAOI and others 12.8%, *P*=.27) nor for rs11140778 (TCA 18.7%, *P*=.64; SNRI or NASSA

SNRS 48.6%, *P*=.58; SSRI 32.4%, *P*=.17; NARI 7.7%, *P*=.34; MAOI and others 12.9%, *P*=.28). Differential data on SNRI and NASSA prescription rates were available for the subsample of 625 MARS patients, but did not reveal any significant differences depending on the genotype for rs1659412 (SNRI

SNRS 27.7%, *P*=.57; NASSA 29.0%, *P*=.86) nor for rs10868223 (SNRI

SNRS 27.4%, *P*=.11; NASSA 29.1%, *P*=.61) nor for rs11140778 (SNRI

SNRS 27.6%, *P*=.97; NASSA 28.8%, *P*=.06).

To further disentangle possible SSRI-specific effects, we first analyzed the response association separately for those patients that received a SSRI (alone or in combination with other antidepressants; *N*=281) and those without a SSRI (*N*=589). In the SSRI group, we found that the *BDNF* SNPs rs2049046 was the strongest associated SNP, together with the *NTRK2* SNP rs11140778 withstanding correction for multiple testing (*P_corr_*=4.5x10^-3^ and *P_corr_*=6.7x10^-3^, respectively; 16 SNPs tested). In the non-SSRI group, the *NTRK2* SNP rs10868223 was the only significant marker (*P_corr_*=.02; supp. table 3).

Second, a refined analysis on antidepressant monotherapy (at discharge) among the most frequently prescribed drugs in the present sample (TCA, *N*=162; SNRI or NASSA, *N*=253; and SSRI, *N*=215) revealed the only significant association with response for rs2049046 (*P*=2.21x10^-3^; *P_corr_*=.02) and for rs11140778 (*P*=2.38x10^-3^; *P_corr_*=.04; supp. table 4). This effect might have been confounded by higher SSRI prescription rates in the discovery sample (36.7% *vs.* 25.7% *vs.* 28.7%; *Χ^2^* =9.71, *df*=2, *P*=.008, *N*=894) where also rs2049046 was strongly associated with treatment response. We therefore analyzed SSRI-treated (*N*=146) and non-SSRI-treated patients (*N*=252) separately in the discovery sample only. Again, rs2049046 and rs11030094 were the strongest associated SNPs in the SSRI-treated subsample (*P_corr_*=6.9x10^-4^ and *P_corr_*=5.7x10^-3^, respectively). Any other SNP association withstood correction for multiple testing. In the non-SSRI-treated patients, the *NTRK2* SNP rs10868223 showed the strongest association with response, but did not withstand correction for multiple testing (*P*=.01; *P_corr_*=.12), nor did any other SNP in this subsample.

As our medication-specific analysis took into account antidepressant drugs only, we aimed to rule out confounding effect of other psychiatric comedication in the two significantly associated SNPs rs2049046 and rs11140778 (supp. table 4). No significant genotype-dependent prescription rates (at discharge) were found for mood stabilizers, benzodiazepines or lithium for rs2049046 (*P*=.43, *P*=.82, *P*=.84, respectively) and rs11140778 (*P*=.16, *P*=.62, *P*=.86, respectively, analysis in patients with available information only, i.e. MARS sample *N*=647).

To further elucidate gender-specific differences, we tested association for males and females separately in our combined sample. After correction for multiple testing, in males (*N*=397) we found only *NTRK2* SNP rs11140778 to be associated (*P*=3.5x10^-4^; *P_corr_*=.01). Among females (*N*=497), only rs1659412 was significant (*P*=1.25x10^-3^; *P_corr_*=.02; supp. table 5) and no association was observed for rs11140778.
